# The changing 50% inhibitory concentration (IC_50_) of cisplatin: a pilot study on the artifacts of the MTT assay and the precise measurement of density-dependent chemoresistance in ovarian cancer

**DOI:** 10.18632/oncotarget.12223

**Published:** 2016-09-23

**Authors:** Yifeng He, Qiujing Zhu, Mo Chen, Qihong Huang, Wenjing Wang, Qing Li, Yuting Huang, Wen Di

**Affiliations:** ^1^ Department of Obstetrics and Gynecology, Ren Ji Hospital, School of Medicine, Shanghai Jiao Tong University, Shanghai 200127, China; ^2^ Shanghai Key Laboratory of Gynecologic Oncology, Ren Ji Hospital, School of Medicine, Shanghai Jiao Tong University, Shanghai 200127, China; ^3^ State Key Laboratory of Oncogene and Related Genes, Shanghai Cancer Institute, Ren Ji Hospital, School of Medicine, Shanghai Jiao Tong University, Shanghai 200127, China; ^4^ Department of Gynecology, Obstetrics and Gynecology Hospital, Fudan University, Shanghai 200011, China; ^5^ Tumor Microenvironment and Metastasis Program, The Wistar Institute, University of Pennsylvania, Philadelphia, Pennsylvania 19104, USA; ^6^ Children's Research Institute, Children's National Medical Center, Washington DC 20010, USA

**Keywords:** inconsistency, cisplatin, 50% inhibitory concentration, ovarian cancer, density

## Abstract

Inconsistencies in the half-maximal (50%) inhibitory concentration (IC_50_) data for anticancer chemotherapeutic agents have yielded irreproducible experimental results and thus reciprocally contradictory theories in modern cancer research. The MTT assay is currently the most extensively used method for IC_50_ measurements. Here, we dissected the critical reasons behind MTT-dependent IC_50_ inconsistencies. We showed that IC_50_ errors caused by the technical deficiencies of the MTT assay are large and not adjustable (range: 300–11,000%). To overcome severe MTT artifacts, we developed an unbiased direct IC_50_ measurement method, the limiting dilution assay. This detection technique led us to the discovery of the inherent density-dependent chemoresistance variation of cancer cells, which is manifold and unpredictable in its forms. The subsequent intracellular signaling pathway analysis indicated that pAkt and p62 expression levels correlated with alterations in the IC_50_ values for cisplatin in ovarian cancer, providing an explainable mechanism for this property. An *in situ* pAkt-and-p62-based immunohistochemical (IHC_pAkt+p62_) scoring system was thereby established. Both the limiting dilution assay and the IHC_pAkt+p62_ scoring system accurately predicted the primary chemoresistance against cisplatin in ovarian cancer patients. Furthermore, two distinct chemoresistant recurrence patterns were uncovered using these novel detection tools, which were linked to two different forms of density-chemoresistance relationships (positively vs. negatively correlated), respectively. An interpretation was given based on the cancer evolution theory. We concluded that the density-related IC_50_ uncertainty is a natural property of the cancer cells and that the precise measurement of the density-dependent IC_50_ spectrum can benefit both basic and clinical cancer research fields.

## INTRODUCTION

Determination of the half-maximal (50%) inhibitory concentration (IC_50_) is essential for understanding the pharmacological and biological characteristics of a chemotherapeutic agent [1, 2]. Since the invention of a colorimetric technique – the 3-(4,5-dimethylthiazol-2-yl)-2,5-diphenyltetrazolium bromide (MTT) assay – the process used for IC_50_ determination has become easier and can be performed in a 96-well plate [3–5]. In contrast to traditional methods for calculating percentages of killed cells, the MTT assay establishes the cytotoxicity of a compound based on decreases in intracellular NAD(P)H-dependent oxidoreductase activity [3, 4]. The initial status of living cells is defined by the optical densities (ODs) of the control wells, the mean of which is set to a survival rate of 100% (i.e., inhibitory rate of 0%). The concentration corresponding to a survival rate of 50% is defined as the IC_50_ [5]. However, the chromogenic product of MTT is insoluble and must therefore be solubilized prior to spectrophotometric analysis [3]. To avoid this step, several functionally identical tetrazolium dyes that produce soluble formazans, such as 3-(4,5-dimethylthiazol- 2-yl)-5-(3-carboxymethoxyphenyl)-2-(4-sulfophenyl)-2H- tetrazolium (MTS, also called “one-step” MTT) and “water-soluble tetrazolium salts” (WSTs, of which WST-8 is widely used and known as Cell Counting Kit 8 (CCK8)), have been developed [6, 7]. These analogues provide simplified protocols for IC_50_ measurements.

MTT and analogue assays have been used for 30 years in cancer research [3, 6, 7] but rarely yield a consistent IC_50_ value for a given chemical compound against a given cancer cell line. For example, 24-h cisplatin vs. SKOV-3 cells offers IC_50_ values ranging from 2 to 40 μM (Supplementary Materials and Methods S1, Supplementary Tables S1 and S2). This issue was initially attributed to differences among the manufacturers and formulae used by different laboratories. However, we later found that even within the same laboratory, the MTT and analogue assays produce variable IC_50_ values among different staff researchers and between different experimental repeats performed by the same researcher. Therefore, the degree of chemoresistance identified through an MTT assay by one laboratory may not be reproducible and should not be used to depict the pharmacological and biological traits of the cancer cell line [8–10]. We evaluated these differences and noted that control wells, which are used as a basis for the calculations of IC_50_ values, are not fixed but rather present substantial variations, and these variations depend on the initial cell seeding density and the proliferation potential of the cell line. Thus, we adopted several control well-free cell viability assays [propidium iodide (PI)-based apoptosis analyses and trypan blue-based cell counting] to re-measure IC_50_ data. However, these assays did not decrease the density-dependent IC_50_ variation, and this intrinsic chemoresistance instability of cancer cells prompted us to explore the underlying mechanisms and clinical implications of IC_50_ inconsistencies.

In this study, our primary aim was to quantify the density-dependent and density-independent IC_50_ errors caused by MTT analogue assays and to understand the major technical reasons for these errors. To provide precise and unbiased IC_50_ measurements, we developed a novel technique that employs a limiting dilution assay to objectively evaluate the chemoresponsiveness of cancer cells at different seeding densities. Our findings have not only revealed the uncorrectability of the deficiencies in MTT analogue assays but also clarified that the irregular density-dependent variation in chemoresistance is an inherent property of cancer cells. As a secondary aim, we investigated the intrinsic reasons behind the inherent density-dependent chemoresistance variations. The contributions of several well-known chemoresistance-related intracellular signaling pathways were analyzed, and we observed that the density-dependent variations in the intensities of some essential signaling components were correlated with the variations in the chemoresistance of cancer cells. The changing IC_50_ values could be calculated based on the expression levels of these essential components. Furthermore, we assessed the clinical significance of the *in vitro* findings. An *in situ* immunohistochemistry (IHC)-based evaluation tool, denoted the IHC_pAkt+p62_ scoring system, was established and used to predict the primary chemoresponsiveness of cancer patients and their long-term outcomes. Both the limiting dilution assay and the IHC_pAkt+p62_ scoring system achieved diagnostic efficacies superior to that of the MTT assay. The chemical compound and cancer system adopted in this study were cisplatin and ovarian cancer [note: cisplatin is a basic component of the first-line taxol-platinum (TP) chemotherapy for ovarian cancer patients]. The entire research strategy can also be used for exploring the density-dependent IC_50_ variations in other agents and cancer systems and for assessing the chemoresponsiveness of cancer patients based on the derived IHC signatures.

## RESULTS

### Current application of MTT and analogues (MTS and CCK8) for IC_50_ measurements: a literature review

Table 1 depicts the journals selected for our evaluation of the MTT, MTS and CCK8 assays. Of the 20,673 articles published in the past five years, 254 contained “MTT” “MTS” or “CCK8” in their titles and/or abstracts. Because other studies might have used MTT, MTS and CCK8 assays without mentioning this in the corresponding titles or abstracts, we searched five issues of *Cancer Research* and noted that 13.3–22.2% of the studies described the use of MTT, MTS, or CCK8 (Table 1). The examined articles also mentioned two additional colorimetric techniques [ATP and the sulforhodamine B (SRB) assays] [11, 12] (Table 1) and used the survival of untreated cells as the basis for estimating the IC_50_ values [11, 12]. From this perspective, 96-well colorimetric techniques were used in 20.7% (29/140) of the studies in the literature (Table 1). IC_50_ errors due to differences in the proliferation rates and/or enzyme activity of cancer cells were not mentioned in any articles. In addition, only 27.6% (8/29) of the manuscripts reported per-well seeding numbers (i.e., cell densities), and the other articles did not provide such information (Supplementary Table S5).

**Table 1:** Applications of MTT analogue assays (and ATP and SRB assays) reported by articles in six academic journals and in five randomly selected issues of *Cancer Research* Volume 73*

Assays	BMC Cancer (a.n. = 3362)	Cancer (a.n. = 3617)	Cancer Research (a.n. = 3998)	Clinical Cancer Research (a.n. = 3776)	Cancer Letters (a.n. = 2172)	International Journal of Cancer (a.n. = 3748)	Cancer Research (Volume 73)		
							Issue 1 (a.n. = 45)	Issues 9-10 (a.n. = 45)	Issues 17-18 (a.n. = 50)
MTT	199 (3.5)	6 (0.2)	4 (0.1)	35 (0.9)	27 (1.2)	18 (0.5)	8 (17.8)	4 (8.9)	7 (14.0)
MTS	23 (0.7)	2 (0.06)	1 (0.03)	9 (0.2)	1 (0.05)	4 (0.1)	1 (2.2)	2 (4.4)	3 (6.0)
CCK8	2 (0.06)	0 (0)	0 (0)	1 (0.03)	2 (0.09)	0 (0)	1 (2.2)	0 (0)	0 (0)
ATP	-	-	-	-	-	-	0 (0)	2 (4.4)	0 (0)
SRB	-	-	-	-	-	-	0 (0)	0 (0)	1 (2.0)

* The data are presented as the number of articles (%). a.n., total number of articles.

### Different IC_50_ values (measured using the MTT assay) of cisplatin against ovarian cancer cells at different seeding densities

**Figure 1:**
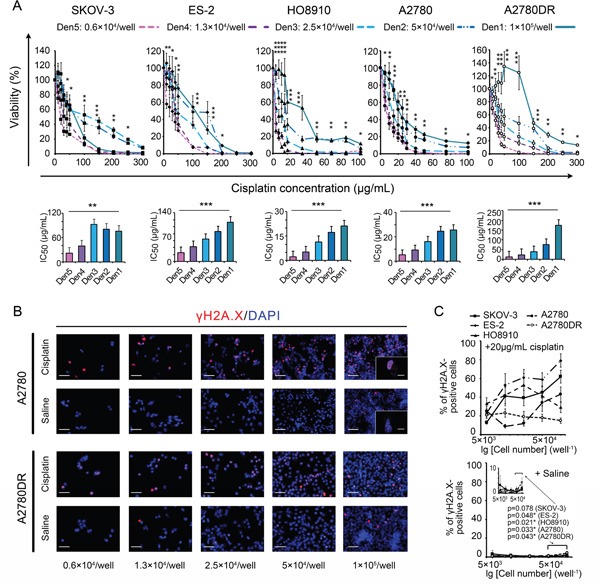
Comparisons of MTT assay-based dose-response curves at different seeding densities **A.** Dose-response curves (upper panel) and corresponding IC_50_ values (lower panel). **B.** Representative cisplatin-induced DNA damage visualized by γH2A.X labelling under a confocal microscope (A2780 and A2780DR are provided as examples). Bars: 50 μm and 10 μm (inset). **C.** Percentage of γH2A.X-positive cells with rescaled inset to show the effect of crowding. *, p < 0.05; **, p < 0.01; ***, p < 0.001, ANOVA (A) and Student's *t*-test (C).

Five ovarian cancer cell lines (see “Materials and Methods”) were used to investigate the effects of seeding density on the MTT-measured IC_50_ values of cisplatin. The A2780DR cell line is a cisplatin-resistant variant of A2780 [13]. MTT data revealed similar IC_50_ patterns for the five cell lines tested, and the IC_50_ values were positively correlated with the seeding densities (Figure 1A). An interpretation of this observation can be linked to the monotonically elevated chemoresistance of cancer cells observed at higher seeding densities. We therefore compared the percentage of γH2A.X (a serine 139-phosphorylated version of H2A.X caused by DNA damage)-positive cells at different cell densities (Supplementary Materials and Methods S2 and Figure S1) [14] and found that the percentage of γH2A.X-positive cells exhibited variations at 24 h post-treatment. However, no corresponding monotonic decreases in the percentage of γH2A.X-positive cells were noted in any of the tested cell lines with the exception of A2780DR (Figure 1B and 1C). The potential density-dependent DNA-protective effect against cisplatin was thereby rejected. Nevertheless, modest but stable increases in the percentage of γH2A.X-positive cells were obtained for the controls at the highest seeding density (2,000 mm^−2^, Figure 1C), reflecting spontaneous apoptosis in over-dense culture conditions.

### Evaluation and analysis of MTT-dependent IC_50_ errors due to uneven proliferation and crowding-induced cell death

**Figure 2:**
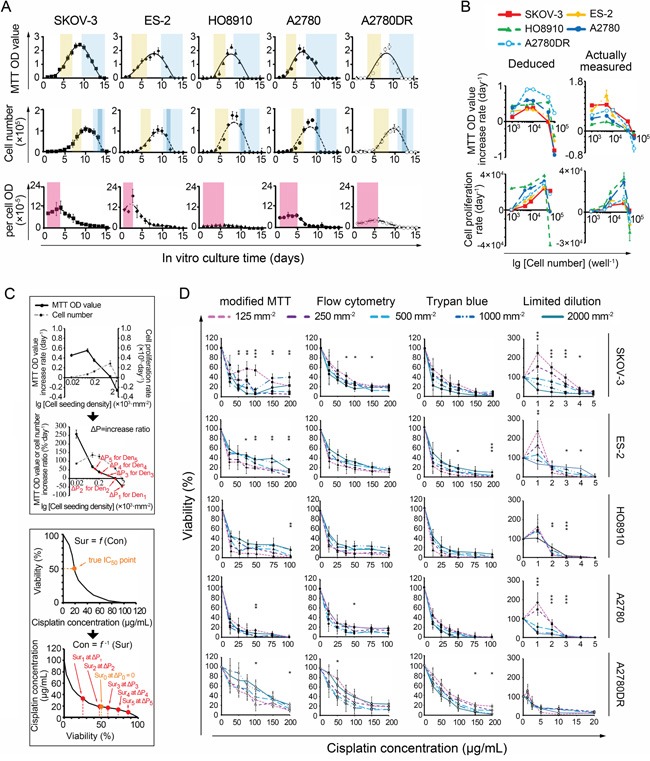
Relationship between IC_50_ values and cell seeding densities **A.** Four stages of MTT- and cell counting-based proliferation curves. Light brown: accelerated growth; blue, reduction; dark blue, crowding-induced cell death; pink: oxidoreductase accumulation. **B.** Deduced and actual cell proliferation rate curves, presented in the forms of MTT ODs (upper panel) and cell numbers (lower panel), respectively. **C.** Theoretical analysis of IC_50_ errors (using A2780 as an example). **D.** Dose-response curves measured by modified and non-MTT IC_50_ assays. *, p < 0.05; **, p < 0.01, ANOVA. (Note, 1 mm^−2^ = 50 well^−1^ in cell number).

The 96-well colorimetric techniques use untreated (control) cells for assessing the initial numbers of treated cells. The control cell numbers change during the observation period, resulting in biases in the calculated IC_50_ values. We therefore recorded cell proliferation curves to estimate the IC_50_ errors. Both the curve obtained using the MTT assay and that obtained by cell counting included slow growth, accelerated growth, a plateau and a reduction in the MTT ODs or cell numbers (Figure 2A). However, the changes in the cell counting-based curves often appeared later than the changes in the MTT OD-based curves. When the cells were slowly increasing, this lag caused an accumulation of oxidoreductase activity (i.e., per-cell MTT OD) and thus defined a “cell content growth” period (Figure 2A and Supplementary Figure S1). At the cell reduction stage, sudden decreases in counts were noted within 24-48 h in over-dense cultures, reflecting cell crowding-induced death (Figure 2A). In accordance with data deduced from proliferation/growth curves, the measured proliferation/growth rate was elevated at lower seeding densities, decreased at moderate densities, and became negative at the highest densities (Figure 2B). These data were used to analyze MTT-dependent IC_50_ errors as follows:

Assuming the relationship between the actual survival rate (Sur) and the concentration of the chemical compound (Con) can be expressed as Sur = f(Con); thus, if Sur = 0.5, Con(50) = f^−1^(0.5) (Equation 1), where Con(50) represents the IC_50_ value. Ideally, we set CON(50) as an unbiased IC_50_. For any initial seeding density Den_i_, the actual survival rate of the cancer cells examined can be expressed as Sur_i_. If the 24-h MTT OD-based increase (daily proliferation rate × 1 day) in the ratio of control cells is ΔP (i.e., MTT OD increment/initial MTT OD, Figure 2C), then the actual Sur_i_ = 0.5(1+ΔP) at the time point when the MTT OD of the treated cells equals 50% of the value obtained for the control wells. According to Equation 1, Con_i_(50) = f^−1^(0.5(1+ΔP)), where Con_i_(50) is the apparent IC_50_. Based on pharmacodynamics, f is usually a monotonic decreasing function [1, 2] and so is f^−1^. Hence, if ΔP > 0 (e.g., at the slow or accelerated proliferation stage), Con_i_(50) < CON(50) = IC_50_. If ΔP < 0 (e.g., at the cell reduction or crowding-induced death stage), IC_50_ = CON(50) < Con_i_(50). Assuming that the MTT-measured IC_50_s at Den_1_-Den_5_ (Den_1_: 2,000 mm^−2^, Den_2_: 1,000 mm^−2^, Den_3_, 500 mm^−2^, Den_4_: 250 mm^−2^, Den_5_: 125 mm^−2^) are Con_1_(50)-Con_5_(50), respectively, according to the monotonic characteristic of ΔP (Figure 2C), the result is Con_5_(50) < Con_4_(50) < Con_3_(50) < Con_2_(50) < Con_1_(50), as illustrated in Figure 1A.

**Figure 3:**
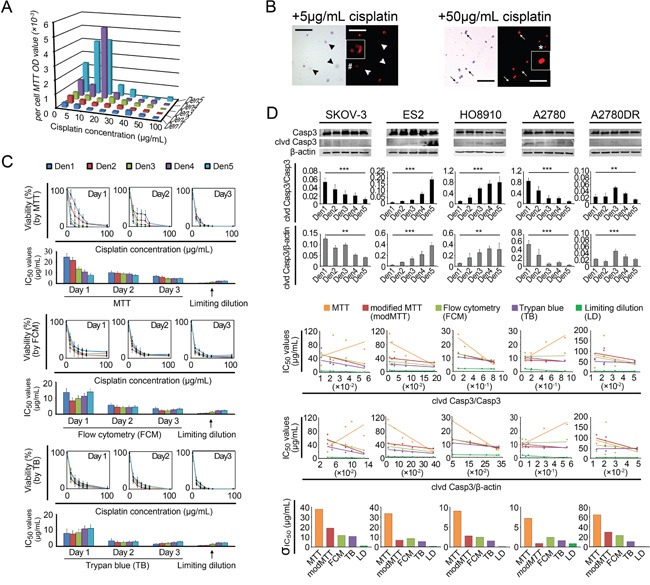
IC_50_ measurement biases **A.** Per-cell MTT ODs measured at different cisplatin concentrations and seeding densities. **B.** Comparison of PI and Trypan blue labeling efficacies. Arrowhead and arrow, PI-negative dead cells. #, karyolysis. *, karyopyknosis. Bar: 200 μm. Inset, 4 × magnification. **C.** Left shift in the IC_50_ value as the non-cisplatin interval was elongated (using A2780 as an example). **D.** Regression analysis between cleaved caspase-3 (clvd Casp3) and IC_50_. Each point shown in rows 4-5 represents a mean IC_50_ value provided by a specific detection method; the standard deviations (σ) are shown in row 6. *, p < 0.05; **, p < 0.01; ***, p < 0.001, ANOVA (D rows 2-3) and Pearson's correlation analysis (D rows 4-5, see Supplementary Table S7).

To reduce ΔP-induced errors, we set the non-cisplatin culture time to 24 h for the control and treated cells in a “modified” MTT assay. Thus, the cell numbers in control wells at the end of the 24-h observation period were equal to the actual numbers of treated cells at the beginning of the 24-h treatment period, and no ΔP would occur (i.e., ΔP = 0). However, the IC_50_ variations decreased but remained present (Figure 2D, Supplementary Table S6). Moreover, a viable cell counting-based analysis indicated that the per-cell MTT ODs varied significantly at different cisplatin concentrations, exhibiting a nonlinear relationship with cell viability. This variability yielded additional IC_50_ errors and induced more IC_50_ variations (Figure 3A). We subsequently performed two non-MTT assays, an FCM-based apoptosis analysis and a trypan blue-assisted cell-counting assay, to confirm the IC_50_ values. However, more inconsistencies in the IC_50_ values were obtained with these two non-MTT systems (Figure 2D, Supplementary Table S6). Microscopic observations revealed that some trypan blue-labeled cells were PI-negative and morphologically in either karyolysis or karyopyknosis (Figure 3B). Furthermore, all IC_50_ data obtained using these three techniques shifted left (decreased) as the intervals between the points of cisplatin withdrawal and the cell-viability measurement were extended, complicating the IC_50_ value calculations (Figure 3C).

Hence, we used a fourth test, the limiting dilution assay. This technique provides a direct count of the cells surviving after cisplatin treatment (Supplementary Materials and Methods S2). The IC_50_ values measured using this assay were significantly lower than those obtained with the previous three assays, even though the inherent density-related variations persisted (Figure 3C). We examined these variations using ratios of cleaved caspase-3/caspase-3 and/or cleaved caspase-3/β-actin (i.e., normalized cleaved caspase-3) obtained through Western blot assays and observed inter-density differences in the cleaved caspase-3 bands (Figure 3D). The linear regression analysis indicated that the cleaved caspase-3/caspase-3 ratios were inversely correlated with the IC_50_ variations for most (SKOV-3, ES-2, HO8910, and A2780DR) of the cell lines tested (Figure 3D and Supplementary Tables S7). These inherent chemoresistance variations substantially contributed to the irregular inter-cell-line IC_50_ relationships. The order of the IC_50_ values for the five cell lines varied depending on the seeding density. For example, SKOV-3, a well-known chemoresistant ovarian cancer cell line, presented chemosensitivity (IC_50_ values similar to those of A2780) at higher seeding densities (1,000–2,000 mm^−2^) and chemoresistance (approximating the IC_50_ values of A2780DR) at lower densities (125-500 mm^−2^), yielding a 6.5-fold change in the IC_50_ values (Supplementary Table S6 and Figure S2).

### Multi-dimensional exploration of potential mechanisms underlying density-dependent chemoresistance

**Figure 4:**
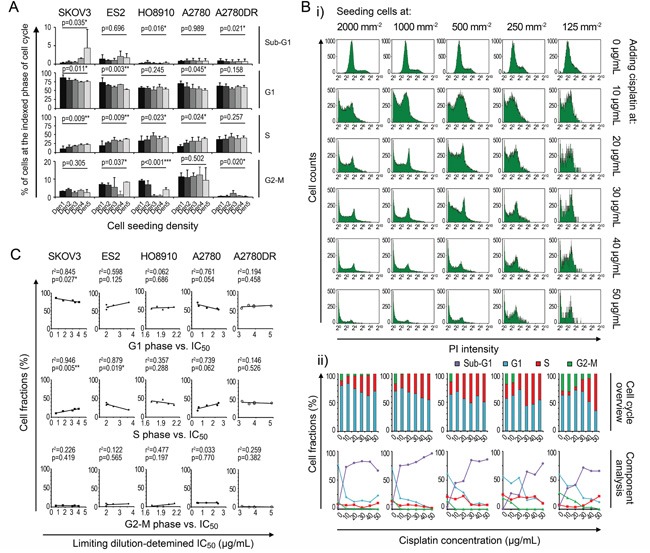
Cell cycle contribution to variation in IC_50_ data **A.** Density-related cell cycle patterns. **B.** Cisplatin-induced changes in the cell cycle (using A2780 as an example). **C.** Linear regression analysis between cell cycle and IC_50_. *, p < 0.05. **, p < 0.01. ***, p < 0.001, ANOVA (A) and Pearson's correlation analysis (C).

To understand the mechanism underlying density-dependent cancer cell chemoresistance against cisplatin, we recorded the cell-cycle patterns at different seeding densities because chemotherapeutic agents are primarily effective against proliferating cells [15]. The FCM analysis showed that the fractions of cells in the G1 and S phases increased and decreased with increasing seeding densities, respectively, whereas no definite patterns were discernible for cells in the G2-M phase (Figure 4A). After cisplatin treatment, the fractions of cells in the G1 and G2-M phases were significantly diminished, whereas the relative fractions of cells in the S phase were slightly decreased or increased (Figure 4B). However, the comparison of the fractions of cells prior to treatment with their corresponding IC_50_ values revealed a significant negative linear correlation, which could support the cell cycle-based theory, only in ES-2 (S-phase fraction vs. IC_50_, Figure 4C). These findings suggest that the proliferation status (e.g., fractions of cells in the S and G2-M phases) minimally influences the density-dependent chemoresistant behavior.

**Figure 5:**
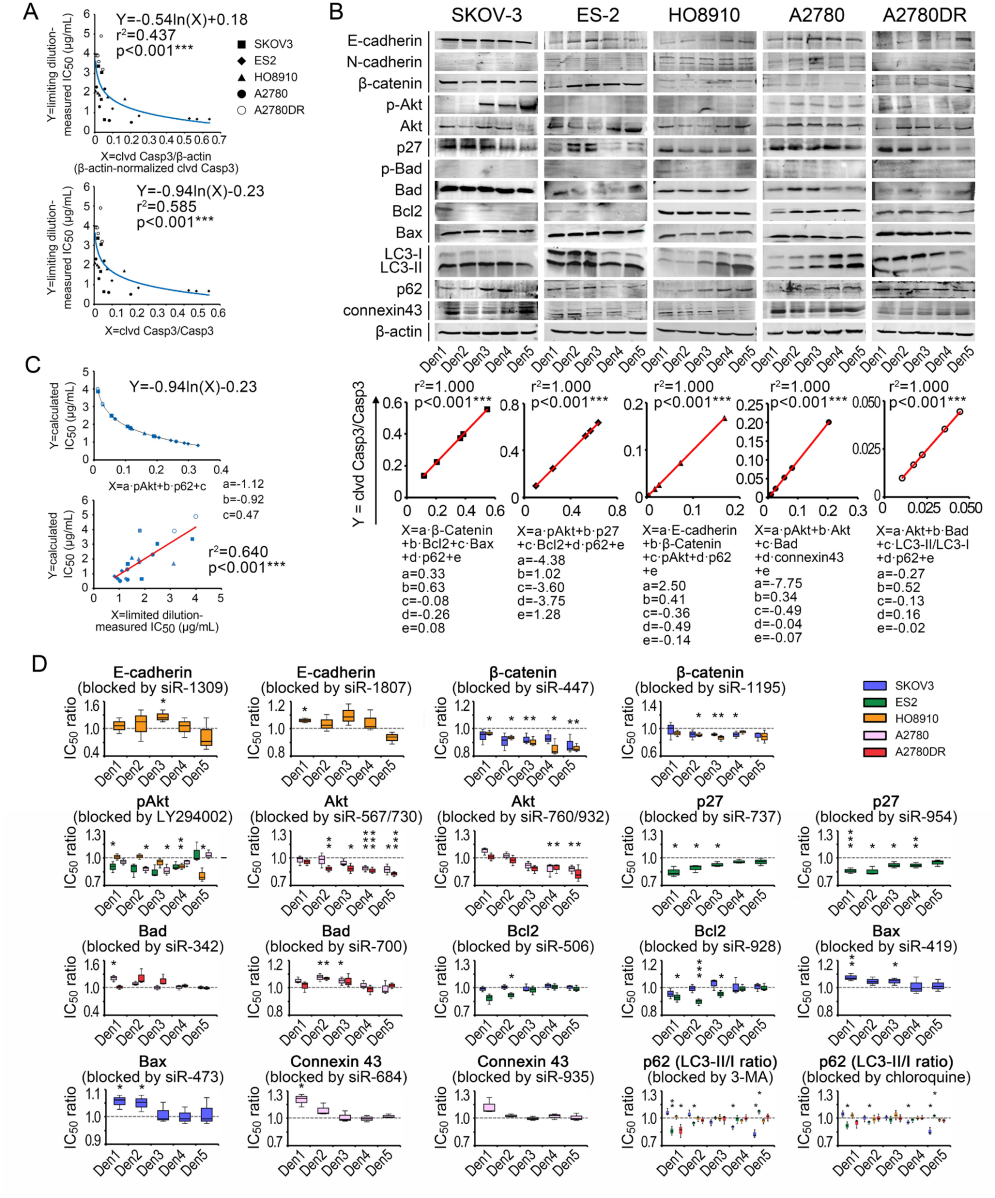
Chemoresistance-related signaling pathway contributions to variation in IC_50_ data **A.** IC_50_-fitting models based on cleaved caspase-3. **B.** Density-related expression patterns of signaling components and fitting formulae. **C.** General IC_50_-fitting formula and validation of its efficacy. **D.** *In vitro* analysis of functions of pathway components in chemoresistance variation. *, p < 0.05. **, p < 0.01. ***, p < 0.001, Pearson's correlation analysis (A–C) and Student's *t*-test (D).

We then analyzed the contributions of several well-known chemoresistance-related signaling pathways, specifically cell-adherence molecules (E-cadherin, N-cadherin, and β-catenin), PI3K-related kinases (pAkt/Akt), apoptosis-related molecules (p-Bad/Bad, Bax, and Bcl-2), cyclin-dependent protein (p27), autophagy-related molecules (LC3b and p62) and gap junction (connexin43)-related pathways [16–27], to the density-dependent IC_50_ variation for cisplatin in ovarian cancer cells (Supplementary Table S8). For our correlation analyses, fitting models for the cleaved caspase-3 ratios, IC_50_ values and seeding densities were established (Supplementary Materials and Methods S2). The cleaved caspase-3/caspase-3 ratios yielded better fits to IC_50_ values in terms of p-values than the normalized cleaved caspase-3 value (Figure 5A). The Western blot assay indicated that basic and density-related expression patterns of each component varied greatly among the cell lines, suggesting the diversity of IC_50_ behaviors (Figure 5B). The multivariate regression analysis indicated that each cell line had a characteristic formula with different necessary components for fitting the cleaved caspase-3 ratios. However, some components, specifically p62 (four times), pAkt (three times), Akt (twice), β-catenin (twice), Bad (twice), and Bcl2 (twice), were applied multiple times, indicating their critical roles in density-dependent chemoresistance (Figure 5B and 5C).

Because regression analyses could only reveal superficial relationships between the pathway components and IC_50_ values of cisplatin, we explored the actual roles of each contributing component. After the administration of siRNAs and small-molecule inhibitors (see Supplementary Table S8 and Figure S3), we demonstrated that β-catenin was able to suppress cisplatin-induced apoptosis in SKOV-3 and HO8910 cells but could not improve chemosensitivity, as the fitting models suggested. This finding also applied to p27 in ES-2, Akt in A2780 and Bad in A2780DR (Figure 5D). In contrast, the accumulation of connexin43 promoted cisplatin-induced apoptosis in A2780 cells, which is the inverse of its role in the fitting formula (Figure 5D). In the cell lines examined, p62 protein was protective against cisplatin at higher pretreatment levels and exerted a cisplatin-sensitizing effect at lower pretreatment levels (Figure 5D). Using these data, we eventually obtained a general IC_50_-fitting formula, in which pAkt and p62 were necessary components, for all five cell lines tested (Figure 5C). The fitting roles of pAkt and p62 were consistent with their functions (Figure 5D) and consistent with the measured IC_50_ values for the five cell lines (p < 0.001, ANOVA, Figure 5C; note that the inter-cell-line pAkt and p62 levels were compared after β-actin-based normalization).

### Clinical implications of density-dependent variations in the IC_50_ values of cisplatin in patients with ovarian cancer

Our data suggest that the MTT assay might not be suitable for IC_50_ measurements and that irregular density-dependent chemoresistance variations of cancer cells are important to consider. Additionally, the use of an intracellular signaling pathway analysis of pAkt and p62 for assessing chemoresponsiveness is worth scrutiny. Cisplatin is a first-line treatment for ovarian cancer; thus, we enrolled 112 ovarian cancer subjects treated with TP chemotherapy, studied their chemoresistant recurrence behaviors and correlated these to cancer cell densities (for clinicopathological characteristics, see Supplementary Table S9).

**Figure 6:**
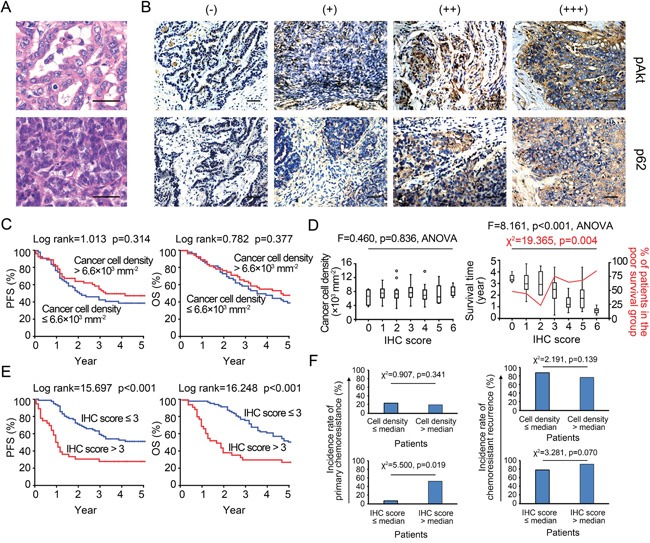
IHC_pAkt+p62_ scoring and survival analysis **A.** Representative microscopic images of density variations in cancer cells among specimens. Bar: 50 μm. **B.** IHC_pAkt+p62_ scoring system. Bar: 50 μm. **C.** Kaplan-Meier analysis (high vs. low density). **D.** Relationships among cancer cell density, survival and IHC_pAkt+p62_ scores in 112 retrospectively enrolled ovarian cancer patients. **E.** Kaplan-Meier analysis (high vs. low IHC_pAkt+p62_ scores). **F.** Incidence rates of primary chemoresistance and chemoresistant recurrence in patients with different cancer cell densities and IHC_pAkt+p62_ scores.

We first assessed the effect of cancer cell density on patient survival. The observed densities of cancer cells ranged from 2.3 × 10^3^ mm^−2^ to 1.3 × 10^4^ mm^−2^ (mean: 6.5 × 10^3^ mm^−2^, SD: 2.2 × 10^3^ mm^−2^, Figures 6A and 6B), much greater than the maximal density mimicked in 96-well plates (i.e., 2 × 10^3^ mm^−2^). Low-density areas were mostly observed in cancer tissue with an abundance of cystic space or at the border where the cancer nest invades into the surrounding normal tissue. Based on the median density (6.6 × 10^3^ mm^−2^), patients were classified as high- or low-density, and no differences in five-year progression-free survival (PFS) or overall survival (OS) were noted (Figure 6C), implying an irregular relationship between cancer cell densities and IC_50_ values *in vivo*. We then established an IHC_pAkt+p62_ scoring system for assessing the chemoresponsiveness of cancer cells individually (Supplementary Table S4). As expected, the IHC_pAkt+p62_ scores did not correlate with cell densities, reinforcing the existence of an irregular relationship between cancer cell density and chemoresistance (Figure 6D). However, when patients were divided into poor (≤ 5 years) and extended (> 5 years) survival groups, poor survival was linked to greater IHC_pAkt+p62_ scores (Figure 6D). Patients with an IHC_pAkt+p62_ score greater than 3 (median = 3) experienced impaired PFS and OS compared with patients with a score less than or equal to 3 (Figure 6E).

Moreover, an analysis of patients with IHC_pAkt+p62_ scores greater than 3 revealed that these patients suffered a significantly higher incidence rate of primary chemoresistance (for the definition, see Supplementary Materials and Methods S2) after a standard course of post-surgical TP chemotherapy (Figure 6F); additionally, more chemoresistance events were observed when TP chemotherapy was re-applied to the recurrence sites in this group (Figure 6F). Chemoresistant recurrence (for the definition, see Supplementary Materials and Methods S2) occurred mainly in the form of remote metastases (liver, spleen, and lung) in low-density cases with IHC_pAkt+p62_ scores greater than 3, whereas *in situ* (peritoneal) cancer recurrence was more frequently found in high-density cases (Supplementary Figure S4). Multivariate analyses of a panel of clinical (e.g., stage, histotype, grade, metastatic status, and residual site) and microenvironmental factors (e.g., stromal cell, M1/M2 macrophage, and CD4^+^/8^+^ T cell densities) indicated that the IHC_pAkt+p62_ score is an independent predictor of long-term survival (PFS and OS) and density-related recurrence patterns (“*in situ*” and “remote”) in our set of 112 retrospective cases (Supplementary Tables S10–S12).

We then focused on density-dependent chemoresistance variations within each cancer specimen. The intra-specimen relationship between cancer cell densities and IHC_pAkt+p62_ scores was characterized by a linear fitting formula, where the coefficient “a” indicates the trend obtained for the changes in the density-dependent IC_50_ values (Supplementary Figure S5A). The patients were divided into two groups: a ≤ 0 (i.e., the local IHC_pAkt+p62_ score increased as the cancer cell density increased) and a > 0 (i.e., the local IHC_pAkt+p62_ score decreased as the cancer cell density increased). Although the chemoresistant cases (IHC_pAkt+p62_ score > 3) were fairly evenly distributed between the low- and high-density groups (Supplementary Figure S5B), we found that those with an “a” value ≤ 0 were more frequent in the low-density group and that those with an “a” value > 0 were more frequent in the high-density group, and this difference was statistically significant (Supplementary Figures S5C and S5D). Moreover, similar to the cancer recurrence patterns observed in the patients classified to the low- and high-density groups (Supplementary Figure S4 and S5E), the highest rate of *in situ* (intraperitoneal) chemoresistant recurrence occurred in patients with “a” values > 0 and an IHC_pAkt+p62_ score > 3, and the greatest rate of remote metastasis occurred in patients with “a” values ≤ 0 and an IHC_pAkt+p62_ score > 3 (Supplementary Figure S5F).

**Figure 7:**
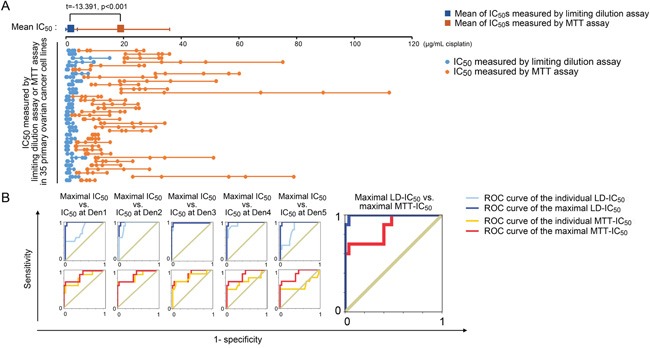
Clinical implications of MTT-IC_50_ and LD-IC_50_ **A.** Comparison between MTT-IC_50_ and LD-IC_50_ spectra in 35 primary ovarian cancer cell lines (Student's *t*-test). IC_50_ values were measured at five seeding densities and are points in each IC_50_ spectrum line. **B.** ROC curves of the maximal and individual MTT-IC_50_ values and LD-IC_50_ values. The ROCs of the maximal IC_50_ values were serially compared with the ROCs of each individual IC_50_ value obtained with each measurement system. The ROCs of the maximal IC_50_ were compared between the MTT and limiting dilution assays.

Direct chemosensitivity cannot be measured in paraffin-embedded specimens, and IC_50_ data are unavailable in retrospective cohort studies. As a result, we enrolled 35 newly diagnosed ovarian cancer subjects (for patient characteristics, see Supplementary Table S13). Primary ovarian cancer cell lines were constructed using isolated cancer cells from patient tumors. Both MTT and limiting dilution assays were performed with each primary cancer cell line at multiple seeding densities (i.e., Den_1_ -Den_5_). The observed IC_50_ values varied with the cell density (Figure 7A), and the respective IC_50_ spectra measured using the MTT assay (MTT-IC_50_) and the limiting dilution assay (LD-IC_50_) were significantly different across the tested cell lines (Figure 7A and Supplementary Figures S6A – S6C). These differences in IC_50_ measurements followed a similar pattern to the MTT vs. limiting dilution assay differences observed across five established ovarian cancer cell lines (Figure 3C). To compare the use of MTT-IC_50_ and LD-IC_50_ spectra for predicting primary chemoresistance against TP chemotherapy, we used two strategies. First, the prognostic values of each IC_50_ at a given seeding density were analyzed. Next, only the prognostic values of the maximal IC_50_ values within a seeding density spectrum were analyzed. The data indicated that when the maximum LD-IC_50_ was considered predictive, the greatest area under the ROC curve for primary chemoresistance was obtained (Figure 7B). In contrast, neither the maximal MTT-IC_50_ nor individual IC_50_ values at any given seeding density offered satisfactory diagnostic efficacy (Figure 7B). At the maximal Youden's index (Table 2), the sensitivity and specificity of the maximal LD-IC_50_ for predicting primary chemoresistance were excellent; however, the maximal Youden's indices for the maximal MTT-IC_50_ and individual IC_50_ values yielded insufficient sensitivity and specificity compared with that of the maximal LD-IC_50_ (Table 2).

**Table 2:** Diagnostic performance of MTT-IC_50_ values and LD-IC_50_ values for predicting primary chemoresistance in 35 newly diagnosed ovarian cancer patients

Indexes	Area under the ROC curve	Youden's index_max_	Sensitivity*	Specificity*	PPV*	NPV*
**MTT assay:**						
IC_50_ at Den1	0.808	0.56	0.60	0.96	0.86	0.86
IC_50_ at Den2	0.848	0.70	0.70	1.00	1.00	0.89
IC_50_ at Den3	0.814	0.62	0.70	0.92	0.78	0.88
IC_50_ at Den4	0.708	0.50	0.50	1.00	1.00	0.83
IC_50_ at Den5	0.592	0.50	0.50	1.00	1.00	0.83
Maximum IC_50_	0.868	0.66	0.70	0.96	0.88	0.89
**Limiting dilution assay:**						
IC_50_ at Den1	0.796	0.48	1.00	0.48	0.43	1.00
IC_50_ at Den2	0.952	0.84	1.00	0.84	0.71	1.00
IC_50_ at Den3	0.992	0.96	1.00	0.96	0.91	1.00
IC_50_ at Den4	0.956	0.72	1.00	0.72	0.59	1.00
IC_50_ at Den5	0.818	0.64	1.00	0.64	0.53	1.00
Maximum IC_50_	0.996	0.96	1.00	0.96	0.91	1.00

* These parameters were calculated as the maximal Youden's index was reached. PPV, positive predictive value. NPV, negative predictive value.

Finally, using primary ovarian cancer cell lines, we re-examined the IHC_pAkt+p62_ scoring system for predicting primary chemoresistance and inherent density-dependent variations in the ovarian cancer subjects (i.e., the 35 newly diagnosed). A Pearson's correlation coefficient for the maximal LD-IC_50_ and IHC_pAkt+p62_ scores indicated that the IHC_pAkt+p62_ scores were associated with chemoresistance (Supplementary Figure S6D). We then re-calculated the “a” values (a_LD_s) of the LD-IC_50_ values for each primary cell line in a similar manner to that used for values obtained with the IHC_pAkt+p62_ scoring system (Supplementary Figures S5A and S6B-S6C). A qualitative comparison between the IHC-determined “a” value (a_IHC_) and a_LD_ returned a κ coefficient of 0.598, supporting the consistency between the two methods (Supplementary Figure S6E). However, subsequent quantitative comparisons suggested that the IHC_pAkt+p62_ scoring system is more suitable for qualitative measurements of density-dependent chemoresistance variations in specimens (for a_LD_ vs. a_IHC,_ r^2^ = 0.096, p = 0.074, Pearson's correlation coefficient analysis, Supplementary Figure S6F).

## DISCUSSION

This study revealed that MTT analogue assays have been prevalently (used in approximately 20% of studies) adopted by the modern cancer research community (Table 1). However, the IC_50_ errors (i.e., for cisplatin) caused by MTT assays are large and not adjustable (range: 300–11,000%, Figure 3C and Supplementary Tables S6). Moreover, we confirmed that the density-dependent variation in IC_50_ values for cisplatin is a bona fide natural trait of cancer cells (Figure 2D and 3D). We observed that the behavior of a cancer cell line can vary between chemoresistant and chemosensitive in an unpredictable manner (Supplementary Figure S2) and showed that the expression and modification (e.g., phosphorylation) of intracellular signaling pathways present variations depending on the seeding densities (Figure 5B). Most importantly, the general fitting formula indicates that the density-dependent chemoresistance against cisplatin in ovarian cancer can be estimated using two essential contributing components, specifically pAkt and p62, which allowed us to establish an IHC_pAkt+p62_ scoring system (Figure 6A). The findings from clinical investigations prove the practicality of the limiting dilution assay and the IHC_pAkt+p62_ scoring system for estimating chemoresistance in ovarian cancer (Figures 6 and 7).

Our *in vitro* data indicate three major deficiencies of MTT analogue assays. First, uneven proliferation control can cause systemic errors in IC_50_ measurements (in a broad sense, including all 96-well colorimetric techniques, Figure 2C). Second, irregular changes in oxidoreductase activity can produce a non-linear relationship between MTT ODs and cell viability (Figure 3A), and third, a lack of synchronicity between mitochondrial oxidoreductase decay and cell death can yield artificial elevations in IC_50_ values (Figure 3C). Originally, the MTT assay was not intended for the measurement of IC_50_ values but rather to assess cell viability and metabolic changes in response to exogenous treatment (e.g., lipopolysaccharide, concanavalin A, interleukin 2 and complements) [3]. No linearity was established between MTT ODs and cell viability as external agents were added. In contrast, the innovators suggested that with the administration of certain agents, the per-cell MTT ODs may vary up to seven- or eight-fold [3]. Therefore, this assay was inappropriately adopted for IC_50_ measurements [28]. Among the three deficiencies we identified, the systematic IC_50_ errors caused by uneven proliferation/growth were the most significant in terms of their misleading effects. The purpose of IC_50_ measurements is to identify the dose required for a chemotherapeutic agent to kill 50% of a cell population with a definite initial number of cells. However, in MTT analogue assays, this idea is interpreted as the dose required to inhibit (or kill) 50% of a cell population with an uncertain or changing initial cell number (Figure 2A). Because the ΔP constantly varies, the MTT-measured IC_50_ values will inevitably change (Figure 2B and 2C). Although surrogate techniques, such as FCM- and cell counting-based IC_50_ assays can avert the “control-well” effect, PI and trypan blue labeling are not in synchrony with cancer cell death (Figure 3C). In most indirect viability assays (including the ATP and SRB assays), normal cell activities, such as oxidoreduction, membrane impermeability and ATP synthesis, always cease after a lethal genomic impairment. Therefore, IC_50_ errors are unavoidable. Only the limiting dilution assay, as a direct viability test, ensures that the obtained IC_50_ data closely approximate actual cancer cell survival (Figure 3D). This technique may be more broadly applied, whereas the MTT and its analogue assays need be restricted to the comparison of cancer cell chemoresponsiveness under identical drug-concentrations, seeding densities and culture conditions. Even then, these assays may not provide an accurate IC_50_ value (Figure 3D and 7A).

The inherent density-dependent variation in IC_50_ values for cisplatin has also been described previously [27, 29, 30], but the data are contradictory. For example, Dimanche-Boitrel's group reported the HT29 colon cancer cells exhibit confluence-induced cisplatin-resistant behavior, whereas Jensen's [27] and Fan's groups [30] suggested that the cisplatin sensitivity of two other mammalian cancer cell lines is significantly improved at confluence. Our data, which were derived after eliminating MTT artifacts and using multiple cell lines, indicate that density-dependent chemoresistance varies with density gradients (ES-2/HO8910), against them (SKOV-3/A2780), or bidirectionally (A2780DR). This manuscript may represent the most inclusive description of this issue to date. Regarding the underlying mechanisms, Dimanche-Boitrel's [29] and Itamochi's groups [31] proposed that an increased number of resting (non-proliferating) cells may contribute to elevated chemoresistance at higher seeding densities. However, although our FCM analysis supports the hypothesis that dividing cells (G2-M phase) are major targets of cisplatin [32–34], we could not verify that the cell cycle plays a central role in the density-dependent variation in IC_50_ values (Figure 4B and 4C). Wu's group explained this phenomenon from the perspective of density-related apoptosis and autophagy [35]. Similarly, we examined intracellular signaling pathways linked to apoptosis (Bcl2, Bad, and Bax) and/or autophagy (LC3 and p62), both directly and indirectly (cell-adherent molecules, pAkt/Akt, p27, and Cx43), and found unpredictability with respect to the activities of these pathways and cell densities. These data oppose the findings reported by Wu and colleagues [35]. The apoptotic and autophagic pathways analyzed in the present study are also frequently mentioned in the literature as critical modulators of chemoresistance but have yet to be incorporated into a unified system for quantitative analysis [16–27]. The regression analyses applied in this study serve as a mathematical tool that bridges the components and IC_50_ variations in the cancer cell lines tested. The ratios of cleaved caspase-3/caspase-3 and cleaved caspase-3/β-actin acquired statistical significance in the general fitting models (Figure 5A), although the former was superior, suggesting that the activation of caspase-3 is more indicative of chemosensitivity. The obtained density-related IC_50_-fitting formula was concise and involved two elements, pAkt and p62 (Figure 5B and 5C). pAkt is known to be important in the PI3K pathway, which is responsible for cell proliferation and anti-apoptotic activity [36], whereas p62 is a key indicator of autophagy that functions in protein clearance, NF-κB signaling, and programmed cell death [26, 37–41]. The roles of pAkt and p62 and the consistency between their functions and mathematical formulae reinforce the importance of density-dependent apoptotic/autophagic pathways in the observed variations in chemoresistance.

The significance of the clinical investigations performed using 112 retrospective and 35 prospective cases is that they provided a broader platform to expose the disadvantages of MTT assays. Furthermore, the clinical data support the use of a direct cell viability test, specifically the limiting dilution assay, and its derivative, the IHC_pAkt+p62_ scoring system, for assessing chemoresponsiveness in ovarian cancer patients. The aberrantly high IC_50_ values obtained with MTT assays and their inability to gauge chemoresistance in cancer patients (Figure 7A and 7B) confirm that hysteretic oxidoreductase decay artificially elevates the IC_50_ values and that the uneven proliferation/growth of control cells yields profound systemic IC_50_ errors (Figure 2C and 3C). Therefore, MTT-derived IC_50_ values cannot be used as an *in vitro* predictive index for optimized/personalized chemotherapy. Nevertheless, it is worth noting that even among LD-IC_50_ values, only the maximal IC_50_ of an IC_50_ spectrum rather than individual IC_50_ values at different seeding densities offers satisfactory predictive efficacy (Figure 7B). This effect might be explained by the evolution theory of cancer [42, 43]. If density-dependent variation in chemoresistance promotes adaptive evolution, cancer cells will spontaneously attain their optimal density to achieve the greatest resistance against chemo-selective pressure. This adaptability is rooted in the nature of cancer cells and cannot be pre-configured by the observer. In other words, a fixed density cannot be used to measure maximal IC_50_ values, and the best method involves probing the density-dependent IC_50_ spectrum. There are limitations to using LD-IC_50_ data. First, we cannot currently mimic a cancer cell density greater than 2,000 mm^−2^, a density that is frequently observed in clinically collected specimens (Figure 6A), under *in vitro* culture conditions (Figure 2A and Supplementary Figure S1). Second, physiological elements, such as pH, pO_2_ and pCO_2_ [44–46], that can cause IC_50_ variations have not been taken into account in the present version of the “limiting dilution assay”, and these might contribute to biases from mimicking real *in vivo* conditions. Nevertheless, these limitations do not appear to fundamentally influence the accuracy of the limiting dilution assay for predicting the chemoresponsiveness of enrolled patients. Youden's index of the selected parameter, the maximal LD-IC_50_, was satisfactory for predicting primary chemoresistance (Figure 7B and Table 2).

The IHC_pAkt+p62_ scoring system established in this study is based on the fact that cleaved caspase-3/caspase-3 ratios can substitute for LD-derived IC_50_ for assessing cancer cell chemoresistance against cisplatin (Figure 5A and 5B). Although this scoring system cannot pinpoint one IC_50_ value (Figure 7), it serves as a proxy for measuring chemoresistance that bypasses the tedious steps of living cancer cell isolation and building primary cancer cell lines for “tumor chemosensitivity tests”. The statistical consistencies between IHC_pAkt+p62_ scores and maximal LD-derived IC_50_ values and between a_IHC_ and a_LD_ (Supplementary Figures S6D and S6E) suggest that pAkt and p62 are located at signaling convergence points that give rise to various density-dependent IC_50_ patterns in most ovarian cancer cases. The mechanism underlying the distinct patient outcomes of the two intra-specimen chemoresistance variation forms (“a” ≤ 0 vs. “a” > 0) might be similarly attributed to cancer evolution. For example, in the “a” ≤ 0 condition, because only decreased growth density offers additional protection, cancer cells disseminating into the lymph and blood vessels and/or remote organs might most benefit from chemo-selection pressure. In contrast, under the “a” > 0 condition, cells remaining *in situ* and growing densely might best survive cisplatin treatment. Additionally, for most chemoresistant cases, the cancer recurrences in patients with low cancer cell densities were similar to those with an “a” value ≤ 0, and the rates of cancer recurrences in patients with high cancer cell densities were similar to those with an “a” > 0 (Supplementary Figures S5E and S5F). Under the same evolution theory, adaptive survival might be occurring, and cancer cell density heterogeneity might provide a competitive advantage. Thus, if the patient has a remote metastasis, it might be chemoresistant under low cell density, and if the recurrence is *in situ*, it might be chemoresistant under high-density conditions. An additional emphasis for users of the IHC_pAkt+p62_ scoring system is that, in a few cases, e.g., caspase-3 mutations or the existence of inhibitors of apoptotic proteins (IAPs), the cleaved caspase-3/caspase-3 ratios could be uncoupled from the IC_50_ values [47, 48]. Then, chemoresistant cases would have IHC_pAkt+p62_ scores ≤ 3. The pre-screening of caspase-3 mutations or inhibitors of apoptotic proteins might favor a reliable evaluation with this system.

Notably, although the MTT-dependent IC_50_ errors analyzed in this study were focused on the system consisting of cisplatin and ovarian cancer, the obtained knowledge concerning the reasons for the inconsistency in IC_50_ values is of practical importance for many other chemotherapeutic agents and cancer systems. Indeed, regardless of the agents or cancer cell lines involved, the uneven proliferation of the control cells at different seeding densities (i.e., ΔP variation) will definitely yield systemic errors in IC_50_ measurements because all of the MTT analogue assays rely on the OD reads from the control cells for the IC_50_ calculations [49, 50]. Moreover, regardless of the speed at which a chemotherapeutic agent kills cancer cells, the delayed response of the cellular metabolic system (i.e., the oxidoreductase system) to agent-induced cell death will definitely produce an artificially elevated IC_50_ value, unless this cell death is accompanied by an immediate quenching of the oxidoreductase activity. More importantly, the intracellular signaling pathways analyzed for explaining the density-dependent variation in chemoresistance have also been verified by previous studies as effective targets of other agents. For example, the PI3 kinase/Akt signaling pathway is capable of diminishing the taxol-induced apoptosis of ovarian cancer cells [51], and p62 accumulation can confer endometrial cancer cells with increased resistance to oxidative stress and is associated with poor prognosis [52]. Using taxol as a study subject, similar findings (i.e., biased and artificially elevated IC_50_ values) were obtained (data not shown). Therefore, although the exact forms of density-dependent chemoresistance and the detailed characteristics of the inconsistences in IC_50_ data can differ between agents and cancer systems, the technical deficiencies of MTT assays that we have identified and the concept of a density-dependent IC_50_ spectrum possess broad applications in biomedical and pharmacological research areas. The logical deduction system and IC_50_ evaluation criteria we developed for exploring the true chemoresponsiveness of ovarian cancer cells to cisplatin are also tenable for millions of combinations of other agents and cancer cell lines. Nevertheless, some more specific IHC signatures might be developed for optimizing *in situ* chemoresistance evaluations in new investigational settings.

In summary, this study offers a paradigm for analyzing density-dependent variations in IC_50_ values (particularly for cisplatin) in cancer cells and reveals the underlying mechanisms and practical implications of these phenomena. Because artifacts of MTT analogue assays and inherent variations in chemoresistance are unavoidable, the methods that are currently used to evaluate the literature should be changed. Researchers and clinicians should determine whether a reported chemoresistance or anticancer mechanism remains accurate for one density condition when it was obtained using cells at another density. Moreover, direct cell viability tests (e.g., limiting dilution or colony formation assays) should be used. Cells grown at unstable densities should not be used as a control when calculating an IC_50_ value. Researchers should be aware that if any factor (e.g., a gene) alters the proliferative rate of the control cells, even if it has no effect on chemoresistance, the apparent IC_50_ value will be altered (Figure 2C, note that the initial number of control cells is fixed using the process of serial dilution and colony counting in the limiting dilution assay). Previously, Haibe-Kains's group suggested the establishment of an international standard for assigning a definite IC_50_ to each cancer cell line [10, 53] and found that different IC_50_ detection systems generate severe and irreconcilable contradictions in IC_50_ data between large-scale international pharmacogenomic studies involving hundreds and thousands of cancer cell lines [8, 9]. Based on our findings, we hold the same opinion and propose that inconsistencies in IC_50_ values are a natural property of cancer cells rather than a remediable artifact. Therefore, a dynamic view of IC_50_ values and the methods used to detect the density-dependent IC50 spectrum of a cancer cell line (primary or passaged) must be established. This view will benefit patients and the cancer research community as a whole.

## MATERIALS AND METHODS

### Literature review

A search of PubMed for eligible research articles published between Nov. 11, 2009 and Nov. 10, 2014 in six cancer research journals (see “Results”) was performed using the keywords “MTT”, “MTS” or “CCK8”. The results were manually screened to exclude similar acronyms but different meanings. Five issues of Volume 73 of “Cancer Research” were selected by drawing lots. Each research article was reviewed to identify the chemosensitivity-testing methods used.

### Clinical samples

Ovarian cancer specimens from patients diagnosed between January 1, 2002 and December 31, 2008 were collected from Ren Ji Hospital, School of Medicine, Shanghai Jiao Tong University, Shanghai, China. Additionally, patients diagnosed with stage II-IV ovarian cancer between July 1, 2014 and December 31, 2014 at Ren Ji Hospital and Obstetrics and Gynecology Hospital (School of Medicine, Fudan University, Shanghai, China) were enrolled. Surgical samples were collected for to obtain living cancer cells. Informed consent was obtained from each patient or their first-degree relatives. All patients received standardized post-surgical cisplatin-based (i.e., TP) chemotherapy. The medical records of each patient were carefully analyzed (Supplementary Materials and Methods S2). The research protocol was approved by the ethics committee of Ren Ji Hospital.

### Cell culture

The SKOV-3, ES-2, HO8910, and A2780 ovarian cancer cell lines were purchased from the Cell Resource Center, Shanghai Institute of Life Sciences, Chinese Academy of Sciences (Shanghai, China) or American Type Culture Collection (ATCC, Manassas, VA, USA) with short tandem repeat-based authentications. The A2780DR cell lines was a gift from Dr. Yinhua Yu (MD Anderson Cancer Center, Houston, TX, USA). Time- and density-dependent cell proliferation curves were obtained based on day-to-day MTT assay/fixation and the density gradient-seeding method, respectively. Cancer tissues from surgical samples were cut into small pieces (1-2 mm in diameter) and then treated with 0.25 mg/mL collagenase IV for 30 min at 37°C. The obtained cell suspension was centrifuged at 1,000 rpm for 5 min, and the isolated cells were cultured in Dulbecco's modified Eagle medium supplemented with 15% fetal bovine serum. Contaminating fibroblasts were removed by repeated adherence (Supplementary Materials and Methods S2).

### Cisplatin cytotoxicity

Twenty-four hours prior to cisplatin treatment, the cells were seeded in 96-well plates at densities of 10^5^, 5 × 10^4^, 2.5 × 10^4^, 1.3 × 10^4^ and 0.6 × 10^4^ per well. Serially diluted cisplatin (Sigma–Aldrich, St. Louis, MO, USA) was added by medium replacement. After 24 h of treatment (to mimic the 24-h clinical aspect of cisplatin administration), MTT assays were performed according to previously described procedures [3]. For the modified MTT assays, the control wells were not seeded until cisplatin had been administered (Supplementary Materials and Methods S2).

### Flow cytometry (FCM)

Apoptosis was measured with an FITC Annexin V Apoptosis Detection Kit I (BD biosciences, San Jose, CA, USA). Our cell-cycle analysis was performed using a Cell Cycle and Apoptosis Analysis Kit (Beyotime, Nantong, Jiangsu, China). For all analyses, living or 70% ethanol-fixed cells were used as controls (Supplementary Materials and Methods S2).

### Trypan blue-based cell counting

Cisplatin-surviving/killed cells in 96-well plates were identified and counted using 0.4% trypan blue (Sigma-Aldrich) staining (Supplementary Materials and Methods S2).

### Limiting dilution assay

The cells were seeded in 96-well plates and cultured for 24 h. Control cells were detached using 0.25% trypsin, re-seeded in a 1/10 dilution series, cultured for 24 h and fixed in 10% formaldehyde. Test cells were treated with cisplatin for 24 h, and the subsequent procedures were the same as those used for the controls. At the appropriate dilution gradient, the colonies formed by the control and test cells were counted to calculate the IC_50_ values (Supplementary Materials and Methods S2).

### Western blot assay

Cultured cells (10^6^-10^7^) were washed with PBS and lysed with RIPA buffer (Thermo Fisher, Waltham, MA, USA). The protein level was measured with a BCA assay kit (Thermo Fisher), and the proteins were separated by SDS-PAGE and then transferred to polyvinylidene fluoride membranes (Millipore, Billerica, MA, USA). After labeling with primary and secondary (fluorophore-coupled) antibodies (Supplementary Materials and Methods S2), the membranes were scanned using an Odyssey CLx infrared imaging system (LI-COR, Lincoln, NE, USA).

### siRNA and small-molecule inhibition

Target-specific and control siRNAs (Supplementary Table S3) were purchased from GenePharma (Shanghai, China). LY294002 (Selleckchem, Houston, TX, USA), 3-methyladenine (3-MA, Selleckchem) and chloroquine diphosphate (Sigma) were administered according to the manufacturers' instructions (Supplementary Materials and Methods S2).

### Immunohistochemistry (IHC)

Immunohistochemical quantification of pAkt and p62 in cancer specimens was performed according to previously described procedures [54]. Antibody information and criteria for negative and positive staining appear in Supplementary Materials and Methods S2 (for the IHCpAkt+p62 scoring system, see Supplementary Table S4).

### Statistical analyses

Two-sided χ^2^ test/Fisher's exact test and ANOVA/Student's *t*-test were used for nominal and numerical data, respectively. A kappa coefficient was used to estimate the consistency between qualitative datasets, and Pearson's product-moment correlation coefficient was used to estimate linear relationships between quantitative datasets (Supplementary Materials and Methods S2). SPSS 18.0 software (IBM, Armonk, NY, USA) was used to perform the statistical analyses (p < 0.05 was considered significant). All experiments were performed in triplicate unless otherwise indicated.

## SUPPLEMENTARY MATERIALS




