# Supplementary material for: The changing 50% inhibitory concentration (IC50) of cisplatin: a pilot study on the artifacts of the MTT assay and the precise measurement of density-dependent chemoresistance in ovarian cancer
Source: Oncotarget. 2016 Sep 23;7(43):70803–21. doi: 10.18632/oncotarget.12223 (PMC5342590; doi:10.18632/oncotarget.12223)
Supplement: Supplementary file 1 [file oncotarget-07-70803-s001.pdf]

The changing 50% inhibitory concentration (IC_{50}) of cisplatin: a pilot study on the artifacts of the MTT assay and the precise measurement of density-dependent chemoresistance in ovarian cancer

SUPPLEMENTARY MATERIALS

SECTION I – REPORTED IC_{50} VALUES (CISPLATIN VS. SKOV-3)

MATERIALS AND METHODS S1

Literature review

We searched the literature deposited in the PubMed Central (PMC) Free Full-text Archive as of Dec 10, 2014 using the keywords "IC50", "Cisplatin" and "SKOV-3" and obtained 185 research articles. By individually reviewing each article, we identified 17 articles that provided full information regarding their IC_{50} detection methods and exact IC_{50} values. A summary of the distribution characteristics of these 17 articles in terms of their three key parameters, namely the method used for IC_{50} measurement, cell seeding density and cisplatin treatment time, is provided in Table S1. A summary of the reported IC_{50} values (cisplatin vs. SKOV-3) is given in Table S2 based on the cisplatin treatment time and IC_{50} measurement technique. Because Hall et al. indicated that dimethylsulfoxide (DMSO) can inactivate cisplatin, we paid special attention to the articles that have used DMSO to dissolve cisplatin (reference information: Hall MD, Telma KA, Chang KE, Lee TD, Madigan JP, Lloyd JR, Goldlust IS, Hoeschele JD, Gottesman MM. Say no to DMSO: dimethylsulfoxide inactivates cisplatin, carboplatin, and other platinum complexes. Cancer Res. 2014; 74: 3913-22).

Supplementary Table S1: Characteristics of the parameters "IC_{50} measurement method", "seeding density" and "cisplatin treatment time" in the identified 17 articles*

IC_{50} measurement method	Articles(n=17)	Seeding density(mm^{-2})	Articles(n=17)	Cisplatin treatment time(hours)**	Articles(n=17)
MTT	3(17.6)	<1	1(5.8)	1	1(5.8)
MTS	2(11.8)	50	1(5.8)	2	2(11.8)
WST-1	1(5.8)	60	2(11.8)	24	6(35.2)
CCK8	1(5.8)	80	1(5.8)	48	1(5.8)
SRB	7(41.1)	100	4(23.5)	72	4(23.5)
Resazurin	1(5.8)	100-200	1(5.8)	96	1(5.8)
Colony formation	1(5.8)	400	1(5.8)	120	1(5.8)
Cell counting	1(5.8)	Not available	6(35.2)	Not available	1(5.8)

* The data are presented as numbers (%). MTT, 3-(4,5-dimethylthiazol-2-yl)-2,5-diphenyltetrazolium bromide. MTS, 3-(4,5-dimethylthiazol-2-yl)-5-(3-carboxymethoxyphenyl)-2-(4-sulfophenyl)-2H-tetrazolium. WST-1, water-soluble tetrazolium salt-1. CCK8, Cell Counting Kit 8. Resazurin, a redox dye used with the CellTiter-Blue Cell Viability Assay Kit (Promega).

** Some studies used multiple cisplatin treatment times for the IC_{50} measurements. We presented the respective median times as representative treatment times in Table S1. One such case is illustrated in ref. 15 (Table S2).

Supplementary Table S2: Reported IC_{50} values (cisplatin vs. SKOV-3) – classified based on cisplatin treatment time and IC_{50} measurement method

Cisplatin treatment time (hours)*	IC_{50} measurement method	Seeding density(mm^{-2})	IC_{50} value	Reference No.***
1	MTT	50	25 µmol/L	[1]
2	SRB	N.A.*	40.5 µmol/L	[2]
2	Colony formation	<1	10 µmol/L**	[3]
24	MTS	60	50 nmol/L	[4]
24	WST-1	N.A.	7.5 µmol/L	[5]
24	CCK8	60	15 mg/L	[6]
24	SRB	100	2 µmol/L	[7]
24	SRB	100	40 µmol/L	[8]
24	Cell counting	N.A.	2 µmol/L	[9]
48	Resazurin	100	10 µmol/L	[10]
72	MTT	400	7.5 µmol/L	[11]
72	MTS	100	25 µmol/L**	[12]
72	SRB	N.A.	4.6 µmol/L	[13]
72	SRB	N.A.	20 µmol/L	[14]
96	SRB	100	4 µmol/L	[15]
120	SRB	N.A.	18.55 µmol/L	[16]
N.A.	MTT	80	0.5 mg/L**	[17]

* N.A., not available.
** These IC_{50} values were obtained as cisplatin was dissolved in a DMSO-containing solution.
*** References:

1. Muenyi CS, Pinhas AR, Fan TW, Brock GN, Helm CW, States JC. Sodium arsenite ± hyperthermia sensitizes p53-expressing human ovarian cancer cells to cisplatin by modulating platinum-DNA damage responses. Toxicol Sci. 2012; 127:139-149. doi: 10.1093/toxsci/kfs085.
2. O'Neill CF, Koberle B, Masters JR, Kelland LR. Gene-specific repair of Pt/DNA lesions and induction of apoptosis by the oral platinum drug JM216 in three human ovarian carcinoma cell lines sensitive and resistant to cisplatin. Br J Cancer. 1999; 81:1294-1303. doi: 10.1038/sj.bjc.6694381.
3. Rabik CA, Fishel ML, Holleran JL, Kasza K, Kelley MR, Egorin MJ, Dolan ME. Enhancement of cisplatin [cis-diammine dichloroplatinum (II)] cytotoxicity by O6-benzylguanine involves endoplasmic reticulum stress. J Pharmacol Exp Ther. 2008; 327:442-452. doi: 10.1124/jpet.108.141291.
4. Yoon J, Kim ES, Lee SJ, Park CW, Cha HJ, Hong BH, Choi KY. Apoptosis-related mRNA expression profiles of ovarian cancer cell lines following cisplatin treatment. J Gynecol Oncol. 2010; 21:255-261. doi: 10.3802/jgo.2010.21.4.255.
5. Vang S, Wu HT, Fischer A, Miller DH, MacLaughlan S, Douglass E, Comisar L, Steinhoff M, Collins C, Smith PJ, Brard L, Brodsky AS. Identification of ovarian cancer metastatic miRNAs. PLOS ONE. 2013; 8:e58226. doi: 10.1371/journal.pone.0058226.
6. Kim MG, Pak JH, Choi WH, Park JY, Nam JH, Kim JH. The relationship between cisplatin resistance and histone deacetylase isoform overexpression in epithelial ovarian cancer cell lines. J Gynecol Oncol. 2012; 23:182-189. doi: 10.3802/jgo.2012.23.3.182.
7. McKeage MJ, Abel G, Kelland LR, Harrap KR. Mechanism of action of an orally administered platinum complex [ammine bis butyrato cyclohexylamine dichloroplatinum (IV) (JM221)] in intrinsically cisplatin-resistant human ovarian carcinoma *in vitro*. Br J Cancer. 1994; 69:1-7. doi: 10.1038/bjc.1994.1.

(*Continued*)

8. Kandala PK, Srivastava SK. Diindolylmethane suppresses ovarian cancer growth and potentiates the effect of cisplatin in tumor mouse model by targeting signal transducer and activator of transcription 3 (STAT3). BMC Med. 2012; 10:9. doi: 10.1186/1741-7015-10-9.
9. Cole C, Lau S, Backen A, Clamp A, Rushton G, Dive C, Hodgkinson C, McVey R, Kitchener H, Jayson GC. Inhibition of FGFR2 and FGFR1 increases cisplatin sensitivity in ovarian cancer. Cancer Biol Ther. 2010; 10:495-504. doi: 10.4161/cbt.10.5.12585.
10. Hooks SB, Callihan P, Altman MK, Hurst JH, Ali MW, Murph MM. Regulators of G-Protein signaling RGS10 and RGS17 regulate chemoresistance in ovarian cancer cells. Mol Cancer. 2010; 9:289. doi: 10.1186/1476-4598-9-289.
11. L'Espérance S, Bachvarova M, Tetu B, Mes-Masson AM, Bachvarov D. Global gene expression analysis of early response to chemotherapy treatment in ovarian cancer spheroids. BMC Genomics. 2008; 9:99. doi: 10.1186/1471-2164-9-99.
12. Kim KK, Lange TS, Singh RK, Brard L. Lipophilic aroylhydrazone chelator HNTMB and its multiple effects on ovarian cancer cells. BMC Cancer. 2010; 10:72. doi: 10.1186/1471-2407-10-72.
13. Wintzell M, Löfstedt L, Johansson J, Pedersen AB, Fuxe J, Shoshan M. Repeated cisplatin treatment can lead to a multiresistant tumor cell population with stem cell features and sensitivity to 3-bromopyruvate. Cancer Biol Ther. 2012; 13:1454-1462. doi: 10.4161/cbt.22007.
14. Stronach EA, Alfraidi A, Rama N, Datler C, Studd JB, Agarwal R, Guney TG, Gourley C, Hennessy BT, Mills GB, Mai A, Brown R, Dina R, et al. HDAC4-regulated STAT1 activation mediates platinum resistance in ovarian cancer. Cancer Res. 2011; 71:4412-4422. doi: 10.1158/0008-5472.CAN-10-4111.
15. Mistry P, Kelland LR, Abel G, Sidhar S, Harrap KR. The relationships between glutathione, glutathione-S-transferase and cytotoxicity of platinum drugs and melphalan in eight human ovarian carcinoma cell lines. Br J Cancer. 1991; 64:215-220. doi: 10.1038/bjc.1991.279.
16. Halon A, Materna V, Drag-Zalesinska M, Nowak-Markwitz E, Gansukh T, Donizy P, Spaczynski M, Zabel M, Dietel M, Lage H, Surowiak P. Estrogen receptor alpha expression in ovarian cancer predicts longer overall survival. Pathol Oncol Res. 2011; 17:511-518. doi: 10.1007/s12253-010-9340-0.
17. Serebrovskaya EO, Edelweiss EF, Stremovskiy OA, Lukyanov KA, Chudakov DM, Deyev SM. Targeting cancer cells by using an antireceptor antibody-photosensitizer fusion protein. Proc Natl Acad Sci USA. 2009; 106:9221-9225. doi: 10.1073/pnas.0904140106.

SECTION II – SUPPLEMENTARY MATERIALS AND METHODS

MATERIALS AND METHODS S2

Patient follow-up

All of the patients enrolled in this study were regularly followed up. The follow-up duration for each patient was from the first surgery until termination (i.e., patient death). Primary chemoresistance was defined as a ≤50% reduction in the maximal diameter of the residual site or enlargement of this diameter after a standard course of TP chemotherapy. A standard course of chemotherapy consists of six rounds of cisplatin and taxol treatment and is applicable for all histotypes of ovarian cancer. Each treatment round consists of the intravenous administration of 175 mg/m^2 taxol and 100 mg of cisplatin on Day 1 followed by a rest interval from Days 2 through 21. Abdominal and pelvic B ultrasound examinations were performed at the beginning of each round of treatment. Patients with computed tomography (CT)- or positron emission computed tomography (PET)-confirmed recurrence sites were again administered a standard course of TP chemotherapy. If the recurrence site shrank by ≤50% or was enlarged after TP chemotherapy, it was classified as chemoresistant recurrence (secondary chemoresistance).

Cell culture (proliferation/growth curves and proliferation/growth rate curves)

The cells were grown in Dulbecco's modified Eagle medium (DMEM)/F12 (for ES-2, HO8910, A2780, and A2780DR cells, Gibco, Life Technologies, Grand Island, NY, USA) or McCoy's 5A (for SKOV-3 cells, Gibco) supplemented with 15% fetus bovine serum (FBS, Gibco), 100 units/mL penicillin and 100 mg/mL streptomycin (Gibco) in a 5% CO_2 atmosphere at 37°C. To obtain MTT- and cell counting-based proliferation/growth curves, 1×10^3 cells were seeded into six consecutive wells in 96-well plates of two series (15 plates per series). Each day from Days 1 (the day when the cells were seeded was defined as Day 0) through 15, one plate was randomly selected from each series. The cells within the two selected plates were measured using the MTT assay or fixed with 10% formaldehyde and counted with DAPI staining under a microscope with 100× magnification. For the MTT assays, 24 h before the spectrophotometric measurement, a 1/10 dilution series of control cells of the same type were seeded (in triplicate) into wells (0, 10, 10^2, 10^3, 10^4, and 10^5 per well, respectively) adjacent to the aforementioned six consecutive wells to produce a standard curve of MTT OD-based cell numbers. The average per-well MTT OD values of the tested cells were adjusted by the standard MTT OD-based cell number curves for each plate. To obtain cell counting-based proliferation/growth curves, the fixed cells were stained with 1 μg/mL DAPI and washed twice with phosphate-buffered saline (PBS). The cells in three representative fields were counted under an inverted fluorescence microscope (Nikon Eclipse Ti-E, Melville, NY, USA) to calculate the cell densities. The average cell density from 18 (6×3) microscopic fields was multiplied by the area of the bottom of a well (i.e., 50 mm^2) to obtain the exact number of cancer cells growing over a given timespan (i.e., 24 h). For the deduced proliferation/growth rate curves (MTT- and cell counting-based), the slope (i.e., derivative) of the tangent line cutting the point at a given density (i.e., its abscissa equals an expected density; 1 well^{-1} = 0.02 mm^{-2} in cell number) on a measured proliferation/growth curve was calculated and treated as a new abscissa (i.e., the proliferation/growth rate) to position the point (of which the ordinate is the seeding density) on a deduced curve. Moreover, to assess the actual (MTT- and cell counting-based) proliferation/growth rates, the cells were seeded at expected densities in sextuplicate, and the per-day increments in the MTT ODs or cell numbers were recorded. Two 96-well plates were used for each curve point: one to determine the initial MTT OD values or cell numbers on Day 1, and the other to determine the post-24-h MTT OD values or cell numbers on Day 2. Standard curves for normalizing the measured MTT ODs were obtained using the same method described for the cell-proliferation/growth curves.

Cisplatin cytotoxicity (MTT and modified MTT assays)

Cisplatin (Sigma) was diluted with complete medium (supplemented with FBS and antibiotics) to a series of expected concentrations [note: for each experimental repeat, the cisplatin-containing medium was freshly prepared. The cisplatin powder was allowed to dissolve in the medium at room temperature without any cosolvent, such as dimethyl sulfoxide (DMSO)]. The culture medium in each well was replaced with 100 μL of cisplatin-containing or cisplatin-free medium as appropriate, and the plate was incubated at 37°C for 24 h. For the MTT assays, phenol red-free medium-dissolved MTT (Sigma) was added to each well to a final concentration of 1 mg/mL. Following incubation for 4 h, the media in each well were replaced with 100 μL of dimethyl sulfoxide. The plate was then covered with tinfoil and agitated on an orbital shaker for 15 min. The absorbance at 570 nm was recorded with a filter reference at 620 nm using a Varioskan Flash Multimode Plate

Reader (Thermo Fisher, Waltham, MA, USA). For the modified MTT assays, the control cells were seeded 24 h later in the same 96-well plate in which the treated cells were seeded using the same density gradients as those used for the treated cells. The survival rate of the treated cells (Sur) at a given seeding density (Den) was calculated as follows:

$$\mathbf{Sur}_{\text{Den}} = \frac{\text{average } \mathbf{MTT\ OD} \text{ of treated wells} - \mathbf{Blank}}{\text{average } \mathbf{MTT\ OD} \text{ of control wells} - \mathbf{Blank}} \times \mathbf{100\%}$$

Flow cytometry

The cells were labeled using a FITC Annexin V Apoptosis Detection Kit I (BD biosciences) or a Cell Cycle and Apoptosis Analysis Kit (Beyotime) as appropriate. To determine the fluorescence intensity thresholds of Annexin V-positive or propidium iodide (PI)-positive cells for a flow cytometry assay, untreated living cells were used as controls, labeled with FITC-Annexin and PI dyes (BD biosciences), and detected using consistent parameters (Cytomics FC 500, Beckman Coulter, Brea, CA, USA). All experiments were repeated in triplicate, and the thresholds for positive FITC- and PI-emitting fluorescence were set to the minimums for 99.9% living cells for classification into a double-negative quadrant (i.e., quadrant B3). For each analysis, 10,000-20,000 events were recorded.

Trypan blue-based cell counting

Following cisplatin treatment, the cells in 96-well plates were centrifuged at 5000 rpm for 15 min to allow the dead cells to settle at the bottom of each well. The media were replaced with 0.4% Trypan blue (Sigma), and the plate was incubated at room temperature for 3 min. Images of the cells at the bottom of the wells were obtained for three representative 100× microscopic fields of each well, and the percentages of stained cells (dead cells) were calculated using these images. For every cisplatin concentration gradient, six wells were recorded, and the average percentages of dead cells were used to construct the dose-response curve of cisplatin at a given density.

Limiting dilution assay

The principle of the limiting dilution assay is to apply the concept of the limiting dilution technique in microbiology to establish the initial number of cancer cells at the moment of chemotherapeutic agent administration. The cells used in the limiting dilution assay were first seeded in 96-well plates, as in the MTT assay. After cisplatin administration, the control cells in the 96-well plate were immediately re-seeded in a series of 1/10 dilutions into consecutive wells of a second 96-well plate. Thus, the numbers of cells in the primary wells at the moment of cisplatin administration could be calculated by multiplying the numbers of colonies observed in the re-seeding wells with their corresponding dilution folds. Following 24 h of cisplatin-treatment, the tested cells were re-seeded into a secondary plate, and the control cells were subjected to the same manipulation. The obtained cell numbers would exactly equal the numbers of tested cells at the moment of cisplatin withdrawal. Notably, a key factor in estimating the initial numbers of control/tested cells in the limiting dilution assay is to select a suitable dilution. Excessive dilution could lead to insufficient colony numbers in the wells of the secondary plate and thus to counting biases. For example, if a dilution was close to the order of magnitude of the initial cell number, any >1 miscounts would cause >10% errors in the IC_{50} calculation result because any expected per-well cell colonies under such conditions would not exceed 10; however, a very low re-seeding dilution could also lead to inaccuracies in the IC_{50} measurements because the observed colonies could overlay with one another within the very narrow bottom of a well (the area of a well bottom in a 96-well plate is only 50 mm^2), making it difficult to discern and individually count the colonies. One critical characteristic of the limiting dilution assay is that the measured number of treated cells in each well might exceed the initial seeding number if a low dosage of cisplatin is administered. This result could never be observed in 96-well colorimetric techniques because the number of control cells would increase during the IC_{50} detection period and would not be less than the number of treated cells due to proliferation in the non-cisplatin conditions; however, only the limiting dilution assay can reflect the continued proliferation of cancer cells under low-dose cisplatin conditions.

Western blotting

The antibodies (primary and secondary) used in the Western blotting assay included mouse anti-human β-actin monoclonal antibody (1:500, CW Biotechnology, Beijing, China), rabbit anti-human caspase-3 polyclonal antibody (1:1000, Product No. 9662, Cell Signaling, Danvers, MA, USA), rabbit anti-human E-cadherin polyclonal antibody (1:200, Product No. sc7870, Santa Cruz), rabbit anti-human N-cadherin polyclonal antibody (1:200, Product No. sc7939, Santa Cruz), mouse anti-human β-catenin monoclonal antibody (1:200, Clone No. E-5, Santa Cruz), rabbit anti-human Akt polyclonal antibody (1:200, Product No. ab8805, Abcam), rabbit anti-human pAkt polyclonal antibody (1:1000, Product No. 9271, Cell Signaling), rabbit anti-human p27 polyclonal antibody (1:200, Product No. sc528, Santa Cruz), mouse anti-human Bad monoclonal antibody (1:200, Clone No. C-7, Santa Cruz), goat anti-human p-Bad polyclonal antibody (1:200,

Product No. sc12969, Santa Cruz), rabbit anti-human Bcl2 polyclonal antibody (1:1000, Product No. 2872, Cell Signaling), rabbit anti-human Bax polyclonal antibody (1:1000, Product No. 2772, Cell Signaling), rabbit anti-human LC3b polyclonal antibody (1:000, Product No. 2775, Cell Signaling), rabbit anti-human p62 polyclonal antibody (1:1000, Product No. 5114, Cell Signaling), rabbit anti-human Connexin43 polyclonal antibody (1:200, Product No. sc9059, Santa Cruz), IRDye 680RD goat anti-mouse IgG (1:10000, LI-COR), IRDye 800CW goat anti-rabbit IgG (1:10000, LI-COR), and IRDye 800CW donkey anti-goat IgG (1:10000, LI-COR). The manufacturers' instructions for these antibodies provide citations for the validity of these antibodies in Western blotting assays. The protein molecular markers Chameleon 700 and Chameleon 800 were purchased from LI-COR Biosciences. The relative intensities of the blot bands were determined using ImageJ 1.45s software (National Institute of Health, Bethesda, MA, USA)

siRNA and small-molecule inhibitor blockage

Lipofectamine 2000 (Life Technologies) was used in all siRNA-transfection experiments according to the manufacturer's instructions. To optimize the siRNA sequences and levels for the knockdown of target genes (TGs), a reverse-transcription (RT) quantitative polymerase chain reaction (qPCR) was conducted using a CellAmp Whole Transcriptome Amplification Kit (TaKaRa, Otsu, Shiga, Japan) and a SYBR Premix Ex Taq II (Tli RNase H Plus) ROX plus Kit (TaKaRa). To determine the knockdown effect (i.e., inhibitory rate), the relative expression levels (rE) of the TGs (normalized by GAPDH) were established by qPCR and compared across conditions. The inhibitory effect of each siRNA was calculated as follows:

$$\textbf{siRNA inhibitory effect} = \frac{\mathbf{rE_{TG-siR}}}{\mathbf{rE_{TG-BL}}} = 2^{(\mathbf{Ct_{TG-BL}} - \mathbf{Ct_{GAPDH-BL}}) - (\mathbf{Ct_{TG-siR}} - \mathbf{Ct_{GAPDH-siR}})}$$

where Ct is the cycle threshold for quantifying the amplification product of a specific gene with the qPCR system; TG-BL represents the TG mRNA product from blank control (BL) samples (treated with saline); TG-siR represents the TG mRNA product from siRNA-treated samples; GAPDH-BL represents the GAPDH mRNA expressed in blank control samples; and GAPDH-siR represents the GAPDH mRNA expressed in siRNA-treated samples. The rE level of the TGs in the BL group was set to 1. All primer pairs and siRNA sequences are listed in Table S3 except for the PCR primer pair for GAPDH and the negative control (NC) siRNA, which are the following:

GAPDH-F: 5'-AGCCACATCGCTCAGACAC-3';
GAPDH-R: 5'-GCCCAATACGACCAAATCC-3'.
NC siRNA (5'→3'): UUCUCCGAACGUGUCA
CGUTT and ACGUGACACGUUCGGAGAATT

The small-molecule inhibitors LY294002, 3-methyladenine (3-MA) and chloroquine diphosphate (CQ) were added at final concentrations of 20 μM, 10 mM, and 100 μM, respectively, in plate wells and incubated with the cultured cells for 24 h in 5% CO_2 at 37°C before further processing. Their inhibitory effects on components (pAkt/Akt and LC3/p62) of their target signaling pathways were validated by Western blotting.

Immunohistochemistry (IHC)

For immunohistochemical analyses, 4-μm-thick tissue sections were cut, mounted onto glass slides, dewaxed using xylene and rehydrated through a gradated series of alcohol solution. Antigen retrieval was conducted by heating in a microwave at medium-high temperature for 15 min and a medium-low temperature for 15 min followed by incubating at room temperature for 2 h. Mouse anti-human pAkt monoclonal antibodies (1:50, Clone No. 18F3.H11, Abcam, Cambridge, MA, USA), p62 monoclonal antibodies (1:50, Clone No. ab56416, Abcam) and horseradish peroxidase (HRP)-conjugated goat anti-mouse IgG polyclonal antibodies (1:100, Zhongshan, Beijing, China) were used for the labeling of pAkt and p62 in ovarian cancer specimens. The signal was developed using 3,3'-diaminobenzidine tetrahydrochloride (DAB, Zhongshan). The cell nuclei were counterstained using hematoxylin. The expression levels of pAkt and p62 were semi-quantitatively evaluated as the percentage of cytoplasm-stained (weakly stained and nuclear/membrane-stained cells were excluded) cancer cells in 20-30 400× microscopic fields, and the cells were then classified as one of the following four groups: <25% (negative), 25-50% (weakly positive), 50–75% (moderately positive) and >75% (strongly positive). Each specimen was independently reviewed by two pathologists, and inconsistent results were discussed in a face-to-face consultation. To calculate the cancer cell, stromal cell and immune cell ($CD68^+CD163^+$ and $CD68^+$/HLA-DR^+ macrophage and $CD3^+$/$CD4^+$ and $CD3^+$/$CD4^+$ T cell) densities under a microscope, rabbit anti-human pan-keratin polyclonal antibodies (1:50, Clone No. ab190625, Abcam), CD68 polyclonal antibodies (1:50, Clone No. ab125212, Abcam), mouse anti-human CD163 monoclonal antibodies (1:50, Clone No. ab17051, Abcam), HLA-DR monoclonal antibodies (1:50, Clone No. ab20181, Abcam), rabbit anti-human CD3 polyclonal antibodies (1:50, Clone No. ab5690, Abcam), mouse anti-human CD4 monoclonal antibodies (1:50, Clone No. ab846, Abcam), CD8 monoclonal antibodies (1:50,

Clone No. ab33786, Abcam) and HRP-conjugated goat anti-rabbit IgG polyclonal antibodies (1:100, Zhongshan) and/or an alkaline phosphatase (AP)-conjugated goat anti-mouse IgG polyclonal antibody (1:100, Zhongshan) were applied. All IHC-positive cells were counted in 50×50-μm^2 microscopic fields at 400× magnification, and the mean density of 20-30 randomly sampled cancer areas was used as the general cancer cell density of that specimen.

Statistical analysis

Two-sided Student's t tests were used to compare the rate of γH2A.X-positive cells between the treated cells seeded at densities of 1000 mm^{-2} and 2000 mm^{-2} and the expression levels of target genes between cells transfected with the negative-control siRNA and the target-specific siRNA. ANOVA was used to compare the IC_{50} values that were measured at different densities using different assays, the rate of γH2A.X-positive cells among cells grown at different seeding densities, the protein expression levels of markers in cells grown at different seeding densities, the cell cycle data among cells grown at different seeding densities and the patient survival statuses among patients with different $IHC_{pAkt+p62}$ scores. Two-sided χ^2 tests were used to compare patients with different ages, gravida and para values, menopausal statuses, ascites, peritoneal and lymphatic metastases, histotypes, stages, grades, sizes of residual sites, densities of cancer cells, stromal cells, immune cells (i.e., macrophages, T cells) and $IHC_{pAkt+p62}$ scores. Kaplan-Meier analyses were used to compare the 5-year survival statuses (progression-free survival and overall survival) among patients with different $IHC_{pAkt+p62}$ scores or cancer cell densities. Multivariate Cox regression analyses were performed to assess the independency of clinical and pathological parameters. For overall and progression-free survival analyses, the associations of demographic and clinicopathological characteristics with time to death from ovarian cancer were initially evaluated with a univariate Cox regression model, and those with a significance of $p<0.05$ were then included in the multivariate analysis. Pearson's correlation coefficients were used to analyze the linearity between IC_{50} values and cleaved caspase 3 levels, between IC_{50} values and cell cycle data, between expected (calculated) and measured levels of cleaved caspase 3, and between expected (calculated) and measured IC_{50} values. The IC_{50} values were determined using either the dose-response curve function in Origin 8.0 (OriginLab, Northampton, MA, USA) or the multinomial fitting function in Excel 2013 (Microsoft, Redmond, WA, USA). To construct IC_{50}-fitting models, the linear regression function of SPSS 18.0 software (IBM, Armonk, NY, USA) was applied.

Supplementary Table S3: Primer pairs (for RT-PCR) and siRNA sequences

See Supplementary File 1

Supplementary Table S4: $IHC_{pAkt+p62}$ scoring system

Target proteins	IHC staining levels	Scores	$IHC_{pAkt+p62}$ scoring system
pAkt	Negative	0	$IHC_{pAkt+p62}$ score =Score(pAkt) + Score(p62)
	Weakly positive	1	
	Moderately positive	2	
	Strongly positive	3	
p62	Negative	0	
	Weakly positive	1	
	Moderately positive	2	
	Strongly positive	3	

SECTION III – LITERATURE REVIEW DATA

Supplementary Table S5: Cell seeding densities used in the 96-well colorimetric IC_{50} measurement assays reported in articles [1-29] from five issues of Volume 73 of "Cancer Research"

Seeding density (mm^{-2})	Number of articles (total=8)	Detection methods	Issue No. (publication date)	Reference No.*, **
25	1	ATP***	9 (May 1)	[1]
40	1	SRB	17 (Sep 1)	[2]
50	2	ATP, MTT	10, 17 (May 15, Sep 1)	[3, 4]
60	1	MTT	17 (Sep 1)	[5]
100	1	MTT	18 (Sep 15)	[6]
150	1	MTS	18 (Sep 15)	[7]
300	1	MTT	1 (Jan 1)	[8]

* References:
1. Crompton BD, Carlton AL, Thorner AR, Christie AL, Du J, Calicchio ML, Rivera MN, Fleming MD, Kohl NE, Kung AL, Stegmaier K. High-throughput tyrosine kinase activity profiling identifies FAK as a candidate therapeutic target in Ewing sarcoma. Cancer Res. 2013; 73:2873-2883. doi: 10.1158/0008-5472.CAN-12-1944.
2. Dai B, Yoo SY, Bartholomeusz G, Graham RA, Majidi M, Yan S, Meng J, Ji L, Coombes K, Minna JD, Fang B, Roth JA. KEAP1-dependent synthetic lethality induced by AKT and TXNRD1 inhibitors in lung cancer. Cancer Res. 2013; 73:5532-5543. doi: 10.1158/0008-5472.CAN-13-0712.
3. Katayama R, Aoyama A, Yamori T, Qi J, Oh-hara T, Song Y, Engelman JA, Fujita N. Cytotoxic activity of tivantinib (ARQ 197) is not due solely to c-MET inhibition. Cancer Res. 2013; 73:3087-3096. doi: 10.1158/0008-5472.CAN-12-3256.
4. Shah N, Jin K, Cruz LA, Park S, Sadik H, Cho S, Goswami CP, Nakshatri H, Gupta R, Chang HY, Zhang Z, Cimino-Mathews A, Cope L, et al. HOXB13 mediates tamoxifen resistance and invasiveness in human breast cancer by suppressing ERα and inducing IL-6 expression. Cancer Res. 2013; 73:5449-5458. doi: 10.1158/0008-5472.CAN-13-1178.
5. Zhang G, He P, Gaedcke J, Ghadimi BM, Ried T, Yfantis HG, Lee DH, Hanna N, Alexander HR, Hussain SP. FOXL1, a novel candidate tumor suppressor, inhibits tumor aggressiveness and predicts outcome in human pancreatic cancer. Cancer Res. 2013; 73:5416-5425. doi: 10.1158/0008-5472.CAN-13-0362.
6. Zhang S, Huang WC, Zhang L, Zhang C, Lowery FJ, Ding Z, Guo H, Wang H, Huang S, Sahin AA, Aldape KD, Steeg PS, Yu D. SRC family kinases as novel therapeutic targets to treat breast cancer brain metastases. Cancer Res. 2013; 73:5764-5774. doi: 10.1158/0008-5472.CAN-12-1803.
7. Mygatt JG, Singhal A, Sukumar G, Dalgard CL, Kaleeba JA. Oncogenic herpesvirus HHV-8 promotes androgen-independent prostate cancer growth. Cancer Res. 2013; 73:5695-5708. doi: 10.1158/0008-5472.CAN-12-4196.
8. Zhang L, Gong C, Lau SL, Yang N, Wong OG, Cheung AN, Tsang JW, Chan KY, Khoo US. SpliceArray profiling of breast cancer reveals a novel variant of NCOR2/SMRT that is associated with tamoxifen resistance and control of ERα transcriptional activity. Cancer Res. 2013; 73:246-255. doi: 10.1158/0008-5472.CAN-12-2241.

** The articles describing the use of 96-well colorimetric IC50-determination assays but that did not provide seeding-density information in the Issue Nos. 1, 9, 10, 17, and 18 of Cancer Research Volume 73 are as follows:

9. Gallo M, Ho J, Coutinho FJ, Vanner R, Lee L, Head R, Ling EK, Clarke ID, Dirks PB. A tumorigenic MLL-homeobox network in human glioblastoma stem cells. Cancer Res. 2013; 73:417-427. doi: 10.1158/0008-5472.CAN-12-1881.
10. Laderach DJ, Gentilini LD, Giribaldi L, Delgado VC, Nugnes L, Croci DO, Al Nakouzi N, Sacca P, Casas G, Mazza O, Shipp MA, Vazquez E, Chauchereau A, et al. A unique galectin signature in human prostate cancer progression suggests galectin-1 as a key target for treatment of advanced disease. Cancer Res. 2013; 73:86-96. doi: 10.1158/0008-5472.CAN-12-1260.
11. Liu YP, Yang CJ, Huang MS, Yeh CT, Wu AT, Lee YC, Lai TC, Lee CH, Hsiao YW, Lu J, Shen CN, Lu PJ, Hsiao M. Cisplatin selects for multidrug-resistant CD133+ cells in lung adenocarcinoma by activating notch signaling. Cancer Res. 2013; 73:406-416. doi: 10.1158/0008-5472.CAN-12-1733.

12. Hasina R, Mollberg N, Kawada I, Mutreja K, Kanade G, Yala S, Surati M, Liu R, Li X, Zhou Y, Ferguson BD, Nallasura V, Cohen KS, et al. Critical role for the receptor tyrosine kinase EPHB4 in esophageal cancers. Cancer Res. 2013; 73:184-194. doi: 10.1158/0008-5472.CAN-12-0915.
13. Chang CL, Hsu YT, Wu CC, Lai YZ, Wang C, Yang YC, Wu TC, Hung CF. Dose-dense chemotherapy improves mechanisms of antitumor immune response. Cancer Res. 2013; 73:119-127. doi: 10.1158/0008-5472.CAN-12-2225.
14. Urdinguio RG, Fernandez AF, Moncada-Pazos A, Huidobro C, Rodriguez RM, Ferrero C, Martinez-Camblor P, Obaya AJ, Bernal T, Parra-Blanco A, Rodrigo L, Santacana M, Matias-Guiu X, et al. Immune-dependent and independent antitumor activity of GM-CSF aberrantly expressed by mouse and human colorectal tumors. Cancer Res. 2013; 73:395-405. doi: 10.1158/0008-5472.CAN-12-0806.
15. Kazerounian S, Gerald D, Huang M, Chin YR, Udayakumar D, Zheng N, O'Donnell RK, Perruzzi C, Mangiante L, Pourat J, Phung TL, Bravo-Nuevo A, Shechter S, et al. RhoB differentially controls Akt function in tumor cells and stromal endothelial cells during breast tumorigenesis. Cancer Res. 2013; 73:50-61. doi: 10.1158/0008-5472.CAN-11-3055.
16. Tyner JW, Yang WF, Bankhead A, Fan G, Fletcher LB, Bryant J, Glover JM, Chang BH, Spurgeon SE, Fleming WH, Kovacsovics T, Gotlib JR, Oh ST, et al. Kinase pathway dependence in primary human leukemias determined by rapid inhibitor screening. Cancer Res. 2013; 73:285-296. doi: 10.1158/0008-5472.CAN-12-1906.
17. Huo S, Ma H, Huang K, Liu J, Wei T, Jin S, Zhang J, He S, Liang XJ. Superior penetration and retention behavior of 50 nm gold nanoparticles in tumors. Cancer Res. 2013; 73:319-330. doi: 10.1158/0008-5472.CAN-12-2071.
18. Beesley AH, Firth MJ, Anderson D, Samuels AL, Ford J, Kees UR. Drug-gene modeling in pediatric T-cell acute lymphoblastic leukemia highlights importance of 6-mercaptopurine for outcome. Cancer Res. 2013; 73:2749-2759. doi: 10.1158/0008-5472.CAN-12-3852.
19. Zhang J, Chen L, Liu X, Kammertoens T, Blankenstein T, Qin Z. Fibroblast-specific protein 1/S100A4-positive cells prevent carcinoma through collagen production and encapsulation of carcinogens. Cancer Res. 2013; 73:2770-2781. doi: 10.1158/0008-5472.CAN-12-3022.
20. Jin L, Wessely O, Marcusson EG, Ivan C, Calin GA, Alahari SK. Prooncogenic factors miR-23b and miR-27b are regulated by Her2/Neu, EGF, and TNF-α in breast cancer. Cancer Res. 2013; 73:2884-2896. doi: 10.1158/0008-5472.CAN-12-2162.
21. Shan J, Dsouza SP, Bakhru S, Al-Azwani EK, Ascierto ML, Sastry KS, Bedri S, Kizhakayil D, Aigha II, Malek J, Al-Bozom I, Gehani S, Furtado S, et al. TNRC9 downregulates BRCA1 expression and promotes breast cancer aggressiveness. Cancer Res. 2013; 73:2840-2849. doi: 10.1158/0008-5472.CAN-12-4313.
22. Wang H, Liu K, Geng M, Gao P, Wu X, Hai Y, Li Y, Li Y, Luo L, Hayes JD, Wang XJ, Tang X. RXRα inhibits the NRF2-ARE signaling pathway through a direct interaction with the Neh7 domain of NRF2. Cancer Res. 2013; 73:3097-3108. doi: 10.1158/0008-5472.CAN-12-3386.
23. Shien K, Toyooka S, Yamamoto H, Soh J, Jida M, Thu KL, Hashida S, Maki Y, Ichihara E, Asano H, Tsukuda K, Takigawa N, Kiura K, et al. Acquired resistance to EGFR inhibitors is associated with a manifestation of stem cell-like properties in cancer cells. Cancer Res. 2013; 73:3051-3061. doi: 10.1158/0008-5472.CAN-12-4136.
24. Li R, Wei J, Jiang C, Liu D, Deng L, Zhang K, Wang P. Akt SUMOylation regulates cell proliferation and tumorigenesis. Cancer Res. 2013; 73:5742-5753. doi: 10.1158/0008-5472.CAN-13-0538.
25. Regan Anderson TM, Peacock DL, Daniel AR, Hubbard GK, Lofgren KA, Girard BJ, Schörg A, Hoogewijs D, Wenger RH, Seagroves TN, Lange CA. Breast tumor kinase (Brk/PTK6) is a mediator of hypoxia-associated breast cancer progression. Cancer Res. 2013; 73:5810-5820. doi: 10.1158/0008-5472.CAN-13-0523.
26. Fang LY, Izumi K, Lai KP, Liang L, Li L, Miyamoto H, Lin WJ, Chang C. Infiltrating macrophages promote prostate tumorigenesis via modulating androgen receptor-mediated CCL4-STAT3 signaling. Cancer Res. 2013; 73:5633-5646. doi: 10.1158/0008-5472.CAN-12-3228.
27. Cai J, Fang L, Huang Y, Li R, Yuan J, Yang Y, Zhu X, Chen B, Wu J, Li M. miR-205 targets PTEN and PHLPP2 to augment AKT signaling and drive malignant phenotypes in non-small cell lung cancer. Cancer Res. 2013; 73:5402-5415. doi: 10.1158/0008-5472.CAN-13-0297.
28. Corbin AS, O'Hare T, Gu Z, Kraft IL, Eiring AM, Khorashad JS, Pomicter AD, Zhang TY, Eide CA, Manley PW, Cortes JE, Druker BJ, Deininger MW. KIT signaling governs differential sensitivity of mature and primitive CML progenitors to tyrosine kinase inhibitors. Cancer Res. 2013; 73:5775-5786. doi: 10.1158/0008-5472.CAN-13-1318.
29. Gironella M, Calvo C, Fernández A, Closa D, Iovanna JL, Rosello-Catafau J, Folch-Puy E. Reg3β deficiency impairs pancreatic tumor growth by skewing macrophage polarization. Cancer Res. 2013; 73:5682-5694. doi: 10.1158/0008-5472.CAN-12-3057.

*** In the literature reports, the ATP content of cancer cells was usually measured using the CellTiter-Glo Luminescent Cell Viability Assay Kit (Promega).

SECTION IV – DENSITY-DEPENDENT IC_{50} SPECTRUM DATA

Supplementary Table S6: IC_{50} data from five ovarian cancer cell lines measured by MTT, modified MTT, FCM, Trypan blue-based cell counting and limiting dilution assays at different seeding densities.*

See Supplementary File 1

Supplementary Table S7: Pearson's correlation coefficient analysis of the linearity between cleaved caspase 3 levels and IC_{50} values measured by MTT, modified MTT, FCM, Trypan blue-based cell counting and limiting dilution assays

See Supplementary File 1

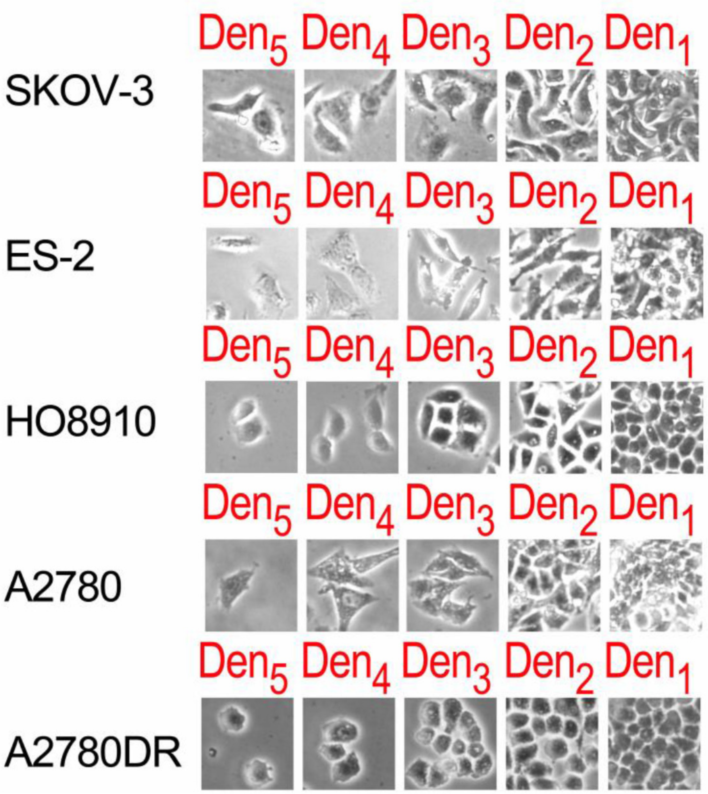

Supplementary Figure S1: Ovarian cancer cell lines cultured at different densities. The images show 200×-magnification microscopic fields of the ovarian cancer cell lines SKOV-3, ES-2, HO8910, A2780 and A2780DR cultured in 96-well plates at five different seeding densities. The observations revealed that the average sizes of the cells cultured at Den4 or Den5 are generally larger than those of the cells cultured at Den1, Den2, and Den3, reflecting a "cell content growth" period. Den1, 2000 mm^{-2}; Den2, 1000 mm^{-2}; Den3, 500 mm^{-2}; Den4, 250 mm^{-2}; Den5, 125 mm^{-2}.

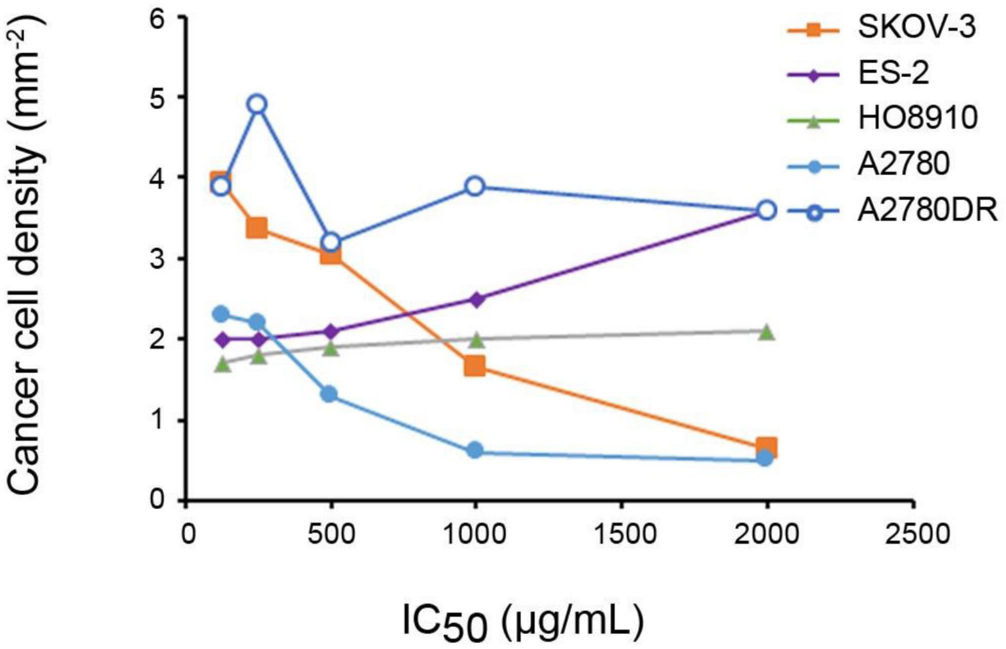

Supplementary Figure S2: Distribution patterns of IC_{50} spectra obtained for five ovarian cancer cells in the Density-IC_{50} two-dimensional space. IC_{50} data for cisplatin with the five cell lines (SKOV-3, ES-2, HO8910, A2780 and A2780DR) were obtained using limiting dilution assays. The comparison reveals that the chemoresistance (i.e., the IC_{50} order) of the five cell lines vary with the seeding densities.

SECTION V – siRNA AND SMALL-MOLECULE INHIBITOR DATA

Supplementary Table S8: RT-PCR-measured inhibitory effects of siRNAs on target gene mRNA levels in ovarian cancer cells seeded at different densities.*

See Supplementary File 1

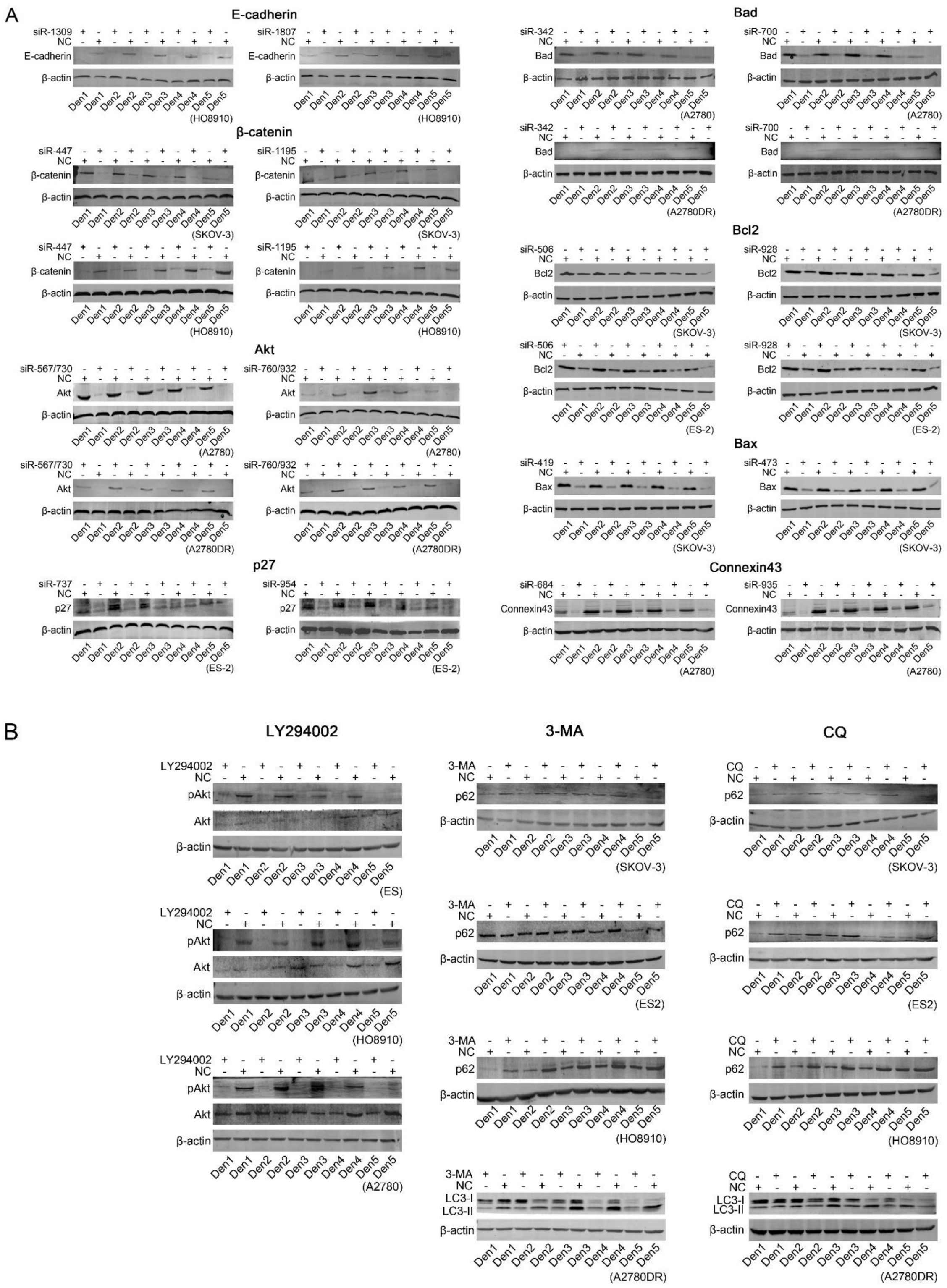

Supplementary Figure S3: Validation of the biological effects of siRNAs and small-molecule inhibitors. A. Western blotting for detecting the expression level of target genes (TGs) in cells transfected with target-specific or control siRNAs. For each TG, two siRNA sequences were applied. **B.** Western blotting for the expression level of TGs in cells treated with small-molecule inhibitors, namely LY294002, 3-MA and CQ. Representative results from three experimental repeats are shown. NC, negative control.

SECTION V – CLINICAL DATA

Supplementary Table S9: Demographic, clinical and pathological characteristics of the 112 previously treated ovarian cancer patients

See Supplementary File 1

Supplementary Table S10: Univariate Cox regression analysis of 5-year progression-free and overall survival in 112 previously treated ovarian cancer patients

See Supplementary File 1

Supplementary Table S11: Multivariate Cox regression analysis of 5-year progression-free and overall survival in 112 previously treated ovarian cancer patients (for characteristics with a p value > 0.05 in the univariate analysis)

Characteristics	Progression-free survivalhazard ratio (95% CI)*	p value	Overall survivalhazard ratio (95% CI)	p value
Peritoneal metastasis		0.591		0.047#
Yes	0.853 (0.483-1.523)		1.926 (1.008-3.676)	
No	1 (reference)		1 (reference)	
Lymphatic metastasis		0.024#		0.051
Yes	2.192 (1.110-4.326)		2.050 (0.997-4.216)	
No	1.000 (reference)		1 (reference)	
Stage		<0.001#		<0.001#
I	0.070 (0.026-0.192)		0.072 (0.022-0.240)	
II	0.118 (0.046-0.306)		0.271 (0.100-0.734)	
III	0.287 (0.133-0.618)		0.435 (0.195-0.975)	
IV	1 (reference)		1 (reference)	
Size of residual site		0.089		0.002#
< 2 cm	0.609 (0.344-1.078)		0.357 (0.184-0.690)	
≥ 2 cm	1 (reference)		1 (reference)	
Density of $CD8^+$ T cells<br>≤ median (3.2 mm^{-2})<br>> median	1.715 (1.100-2.673)<br>1 (reference)	0.017#	2.164 (1.229-3.810)<br>1 (reference)	0.008#
$IHC_{pAkt+p62}$ score<br>≤ median (score = 3)<br>> median	0.462 (0.292-0.729)<br>1 (reference)	0.001#	0.343 (0.193-0.609)<br>1 (reference)	<0.001#

*** CI, confidence interval.**

# Statistical significance: multivariate Cox regression analysis.

Supplementary Table S12: Multivariate Cox regression analysis of 5-year chemoresistant recurrence-free survival in 112 previously treated ovarian cancer patients (for all characteristics).*

See Supplementary File 1

Supplementary Table S13: Clinicopathological characteristics of the 35 newly diagnosed ovarian cancer patients.*

Characteristics	Complete/partial remission** (n = 25)	Primary chemoresistance** (n = 10)	p value#
Age			0.642
< 40	3 (12)	1 (10)	
40-49	4 (16)	1 (10)	
50-59	5 (20)	5 (50)	
60-69	11 (44)	3 (30)	
≥ 70	2 (8)	0 (0)	
Gravidity			0.060
0-1	1 (4)	0 (0)	
2-3	6 (24)	7 (70)	
4-5	12 (48)	3 (30)	
≥ 5	6 (24)	0 (0)	
Parity			0.488
0-1	18 (72)	8 (80)	
2-3	7 (28)	2 (20)	
Menopause			0.155
Yes	11 (44)	7 (70)	
No	14 (56)	3 (30)	
Ascites			0.530
Yes	16 (64)	7 (70)	
No	9 (36)	3 (30)	
Peritoneal metastasis			0.041#
Yes	5 (20)	6 (60)	
No	20 (80)	4 (40)	
Lymphatic metastasis			0.644
Yes	10 (40)	4 (40)	
No	15 (60)	6 (60)	
Histotype			0.389
Serous	21 (84)	9 (90)	
Mucinous	2 (8)	0 (0)	
Endometrioid	1 (4)	0 (0)	
Clear cell	0 (0)	1 (10)	
Undifferentiated	1 (4)	0 (0)	
Stage			0.229
I	0 (0)	0 (0)	
II	5 (20)	2 (20)	
III	18 (72)	5 (50)	
IV	2 (8)	3 (30)	
Grade			0.009#
G1	2 (8)	2 (20)	
G2	4 (16)	6 (60)	
G3	19 (76)	2 (20)	
Size of residual site			N.A.
< 2 cm	0 (0)	0 (0)	
≥ 2 cm	25 (100)	10 (100)	

* Only those patients who were surgically/pathologically diagnosed with stage II-III ovarian cancer and who had residual sites ≥ 2 cm were enrolled and followed up. None of these patients underwent neoadjuvant chemotherapy. The data are presented as numbers (%).

** Complete remission, no evidence of residual site; partial remission, the maximal diameter of the residual site shrank by > 50%; primary chemoresistance, the maximal diameter of the residual site shrank by ≤ 50% or enlarged after a standard course of TP chemotherapy.

Statistical significance: two-sided χ^2 test; for 2×2 tables, Fisher's exact test was applied; N.A., not available.

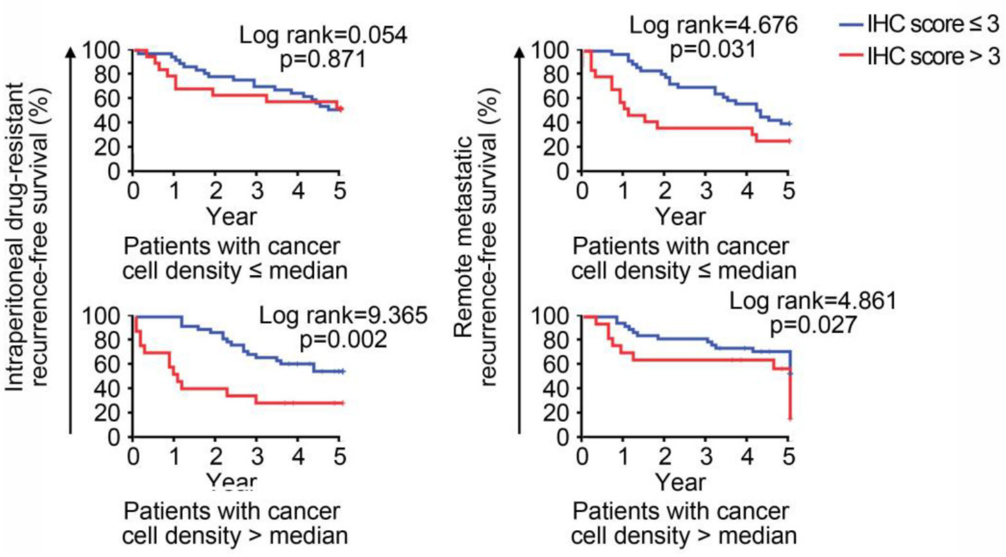

Supplementary Figure S4: Analysis of recurrence patterns in patients with different cancer cell densities and IHC$_{pAkt+p62}$ scores. Kaplan-Meier analyses of 112 retrospective ovarian cancer patients with specimen cancer cell densities ≤ 6600 mm^{-2} and > 6600 mm^{-2}. Patients in the high-density group with higher IHC$_{pAkt+p62}$ scores experienced poorer intraperitoneal chemoresistant recurrence-free survival than those with lower IHC$_{pAkt+p62}$ scores; patients in the low-density group with higher IHC$_{pAkt+p62}$ scores experienced poorer remote metastatic recurrence (chemoresistant)-free survival compared than those with lower IHC$_{pAkt+p62}$ scores.

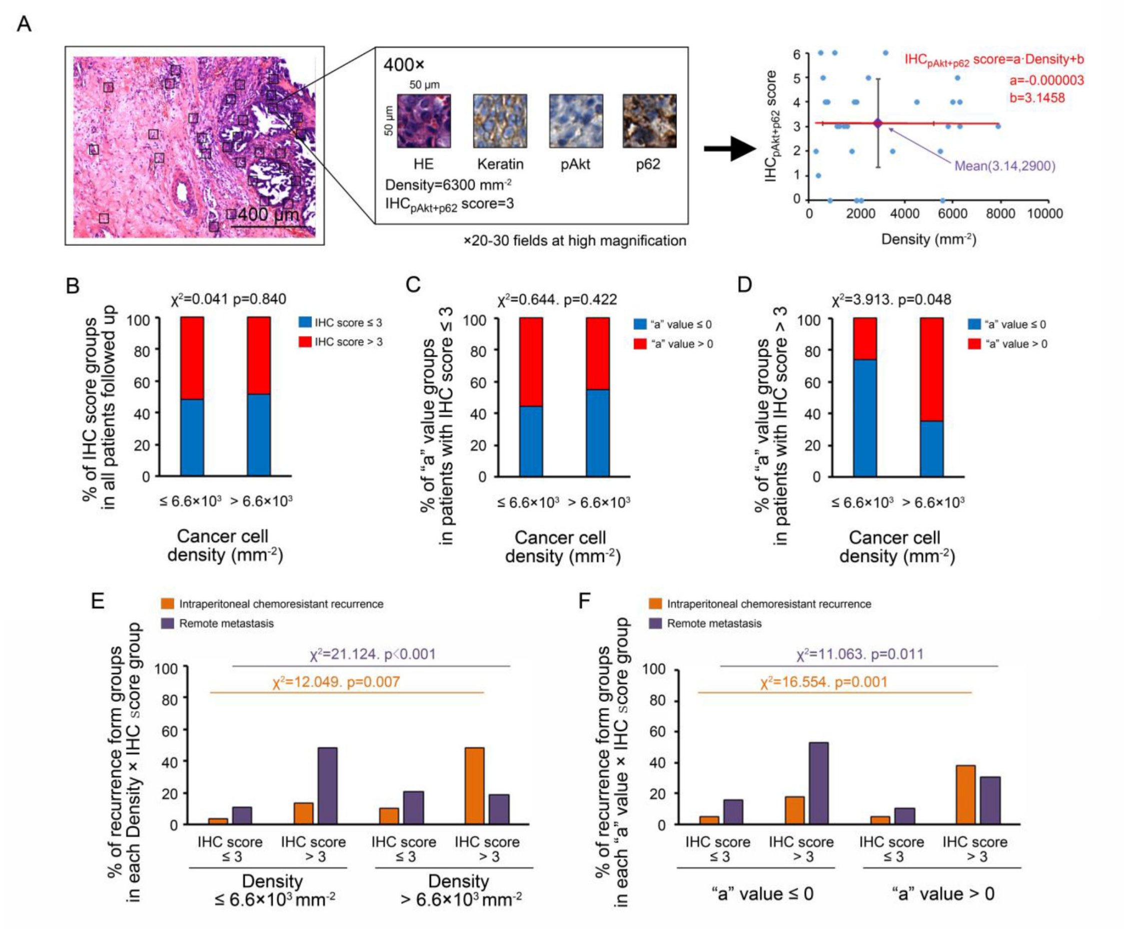

Supplementary Figure S5: Density-dependent and density-independent variations in $IHC_{pAkt+p62}$ scoring and their clinical significance. A. Schematic illustration of the approach used to determine density-dependent variation patterns of $IHC_{pAkt+p62}$ scores for ovarian cancer specimens. The changing trend in the density-dependent $IHC_{pAkt+p62}$ scoring levels was defined by the coefficient of the first-degree term of the linear formula (red line) that was fit to the sampling points ($IHC_{pAkt+p62}$ score vs. density, blue dots) for a specimen (i.e., “a” value). Four serial sections were stained using HE, anti-human keratin antibodies, anti-human pAkt antibodies and anti-human p62 antibodies. The number of cells on a section within the corresponding area of an indicated sampling point was counted, and the mean was used to calculate the representative density value for this point. Any “a” value > 1 indicates that the $IHC_{pAkt+p62}$ staining levels were positively correlated with the cancer cell densities. Otherwise, $IHC_{pAkt+p62}$ scores were negatively correlated with the densities (or a constant in cases where a=0). **B.** Comparison of the characteristics of the density-independent $IHC_{pAkt+p62}$ score distributions in specimens from two cancer cell density groups (low vs. high). **C.** Comparison of the density-dependent variation patterns in $IHC_{pAkt+p62}$ scores for patients with lower $IHC_{pAkt+p62}$ scores (i.e., ≤ 3). **D.** Comparison of density-dependent variation patterns in $IHC_{pAkt+p62}$ scores for patients with higher $IHC_{pAkt+p62}$ scores (i.e., > 3). **E.** Analysis of the distribution patterns of two chemoresistant recurrence forms (i.e., intraperitoneal/*in situ* recurrence and remote metastasis) in patients with various combinations of cancer cell densities and $IHC_{pAkt+p62}$ scores. **F.** Analysis of the distribution patterns of two chemoresistant recurrence forms (i.e., intraperitoneal/*in situ* recurrence and remote metastasis) in patients with various combinations of “a” values and $IHC_{pAkt+p62}$ scores.

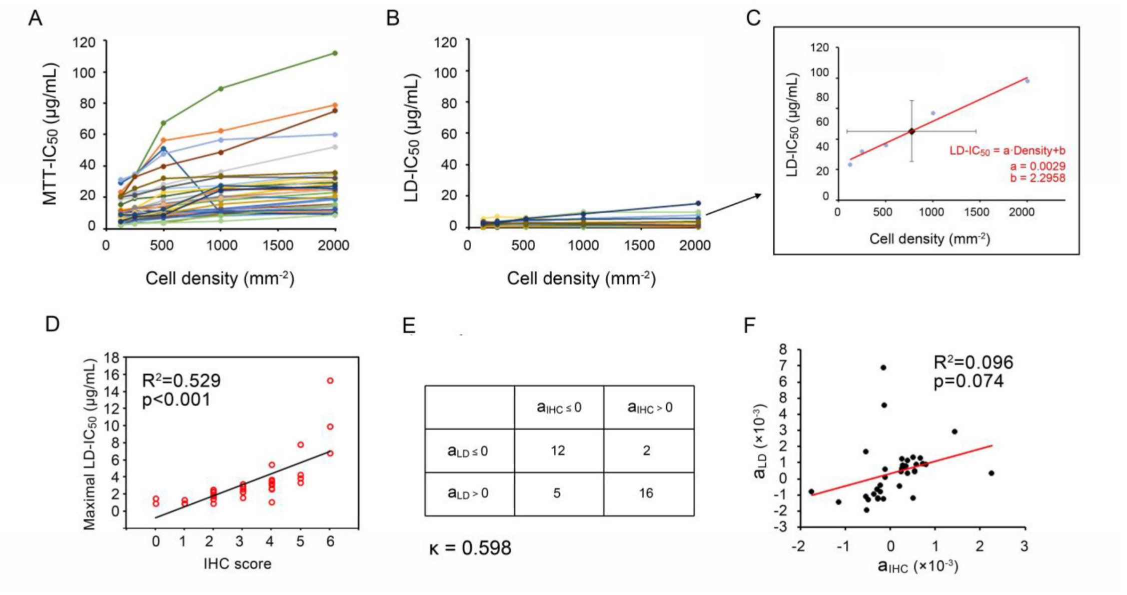

Supplementary Figure S6: Density-dependent IC_{50} variation measured using the MTT assay, $IHC_{pAkt+p62}$ scoring system and limiting dilution assay in 35 newly diagnosed patients. A. Density-dependent IC_{50} variations measured using MTT assays. **B.** Density-dependent IC_{50} variations measured through limiting dilution assays. **C.** Schematic illustration of the approach used for the quantitative characterization of density-dependent variations in IC_{50} data. The changing trend in density-dependent LD-IC_{50} values was defined using the coefficient of the first-degree term of the linear formula (red line), which was fitted to the sampling points (LD-IC_{50} vs. density, blue dots) of a primary cancer cell line (i.e., "a" value). The "diamond" symbol indicates the mean LD-IC_{50} and the density of the sampling points. **D.** Correlation between maximal LD-IC_{50} values and $IHC_{pAkt+p62}$ scores. **E.** Diagnostic consistency between IC_{50} variations ("a" values) defined by the limiting dilution assay and $IHC_{pAkt+p62}$ scoring system, respectively. **F.** Quantitative comparisons between a_{LD} values and a_{IHC} values. Pearson's correlation analysis (D and F) and Kappa consistency analysis (E) were applied.
