# Supplementary material for: The changing 50% inhibitory concentration (IC50) of cisplatin: a pilot study on the artifacts of the MTT assay and the precise measurement of density-dependent chemoresistance in ovarian cancer
Source: Oncotarget. 2016 Sep 23;7(43):70803–21. doi: 10.18632/oncotarget.12223 (PMC5342590; doi:10.18632/oncotarget.12223)
Supplement: Supplementary file 2 [file oncotarget-07-70803-s002.docx]

| **Table S3. Primer pairs (for RT-PCR) and siRNA sequences.** | | | |  | |  | |  |
| --- | --- | --- | --- | --- | --- | --- | --- | --- |
| **Gene symbols** | | **Primer pairs** (5’→3’) | **siRNAs** (5’→3’) **No. 1** | | **siRNAs** (5’→3’) **No. 2** | | | |
| E-cadherin | F | CCATCTGGAGTTTGGTAACGG | siR-1309: | GGUUCAAGCUGCUGACCUUTT | siR-1807: | | CCAGGAGCCAGACACAUUUTT | |
|  | R | CCCACTTTGAATCGGGTGTC |  | AAGGUCAGCAGCUUGAACCTT |  |  | AAAUGUGUCUGGCUCCUGGTT | |
| β-catenin | F | ATCAGTCCTGGGGATCGGAC | siR-447: | GGAUGUGGAUACCUCCCAATT | siR-1195: | | GCUUAUGGCAACCAAGAAATT | |
|  | R | GTGCTCACCCTACTGACGC |  | UUGGGAGGUAUCCACAUCCTT |  |  | UUUCUUGGUUGCCAUAAGCTT | |
| AKT1* | F | TCCTGGTCCTGTCTTCCTCAT | siR-567: | GCUAUUGUGAAGGAGGGUUTT | siR-760: | | GGCCCAACACCUUCAUCAUTT | |
|  | R | GGCAGCCCCTTTGACTTCTT |  | AACCCUCCUUCACAAUAGCTT |  |  | AUGAUGAAGGUGUUGGGCCTT | |
| AKT2* | F | ACAAGGAAAGGGAACCAGCG | siR-730: | GGGCUAAAGUGACCAUGAATT | siR-932: | | CCUCACUGCGCUGAAGUAUTT | |
|  | R | GGTACGCTGTCACCTAGCTC |  | UUCAUGGUCACUUUAGCCCTT |  |  | AUACUUCAGCGCAGUGAGGTT | |
| p27 | F | GTACGAGTGGCAAGAGGTGG | siR-737: | GCCAGCGCAAGUGGAAUUUTT | siR-954: | | GCAUUUGGUGGACCCAAAGTT | |
|  | R | TGCGTGTCCTCAGAGTTAGC |  | AAAUUCCACUUGCGCUGGCTT |  |  | CUUUGGGUCCACCAAAUGCTT | |
| Bad | F | AGACTGAGGTCCTGAGCCGA | siR-342: | CCAGAUCCCAGAGUUUGAGTT | siR- 700: | | GGACUCCUUUAAGAAGGGATT | |
|  | R | CTTCCTCTCCCACCGTAGCG |  | CUCAAACUCUGGGAUCUGGTT |  |  | UCCCUUCUUAAAGGAGUCCTT | |
| Bcl2 | F | GAACTGGGGGAGGATTGTGG | siR-506: | GGGAGAACAGGGUACGAUATT | siR- 928: | | GAGGAUUGUGGCCUUCUUUTT | |
|  | R | CCGTACAGTTCCACAAAGGC |  | UAUCGUACCCUGUUCUCCCTT |  |  | AAAGAAGGCCACAAUCCUCTT | |
| Bax | F | ACAGGGGCCCTTTTGCTTC | siR-419: | CCAGCAAACUGGUGCUCAATT | siR- 473: | | CCAUCAUGGGCUGGACAUUTT | |
|  | R | CTTGGTGGACGCATCCTGAG |  | UUGAGCACCAGUUUGCUGGTT |  |  | AAUGUCCAGCCCAUGAUGGTT | |
| Connexin43 | F | GGCCTTCTTGCTGATCCAGT | siR-684: | GGUAUUGAAGAGCAUGGUATT | siR- 935: | | GGCCUUGAAUAUCAUUGAATT | |
|  | R | GGCGAGAGAGGAAACAGTCC |  | UACCAUGCUCUUCAAUACCTT |  |  | UUCAAUGAUAUUCAAGGCCTT | |
| * To obtain a significant inhibitory effect on the expression of Akt proteins, siRNAs corresponding to two major AKT homologous genes were synthesized. | | | | | | | | |

| **Table S6. IC_50_ data from five ovarian cancer cell lines measured by MTT, modified MTT, FCM, Trypan blue-based cell counting and limiting dilution assays at different seeding densities.*** | | | | | | | | | | | | | | | | | | | | | | | | | |
| --- | --- | --- | --- | --- | --- | --- | --- | --- | --- | --- | --- | --- | --- | --- | --- | --- | --- | --- | --- | --- | --- | --- | --- | --- | --- |
| **Cell lines** | **MTT** | | | | | **Modified MTT** | | | | | **FCM** | | | | | **Trypan blue** | | | | | **Limiting dilution assay** | | | | |
|  | **Den1** | **Den2** | **Den3** | **Den4** | **Den5** | **Den1** | **Den2** | **Den3** | **Den4** | **Den5** | **Den1** | **Den2** | **Den3** | **Den4** | **Den5** | **Den1** | **Den2** | **Den3** | **Den4** | **Den5** | **Den1** | **Den2** | **Den3** | **Den4** | **Den5** |
| SKOV-3 | 67.3 | 93.3 | 108.0 | 31.7 | 20.1 | 24.9 | 33.1 | 23.7 | 37.9 | 70.5 | 24.4 | 41.3 | 35.0 | 43.2 | 55.9 | 15.8 | 18.6 | 22.3 | 25.9 | 43.3 | 0.6 | 1.7 | 3.0 | 3.4 | 3.9 |
|  | ±0.5 | ±0.5 | ±0.5 | ±0.4 | ±0.4 | ±0.5 | ±0.4 | ±0.5 | ±0.4 | ±0.5 | ±0.5 | ±0.4 | ±0.5 | ±0.5 | ±0.5 | ±0.3 | ±0.4 | ±0.5 | ±0.5 | ±0.4 | ±0.1 | ±0.2 | ±0.3 | ±0.3 | ±0.3 |
| ES-2 | 110.9 | 85.9 | 57.8 | 46.3 | 26.0 | 42.0 | 37.0 | 40.5 | 29.0 | 26.9 | 40.5 | 24.1 | 24.0 | 23.6 | 18.8 | 28.5 | 20.1 | 17.1 | 15.9 | 14.9 | 3.6 | 2.5 | 2.1 | 2.0 | 2.0 |
|  | ±0.6 | ±0.5 | ±0.4 | ±0.5 | ±0.4 | ±0.6 | ±0.5 | ±0.5 | ±0.5 | ±0.5 | ±0.5 | ±0.4 | ±0.4 | ±0.5 | ±0.5 | ±0.5 | ±0.5 | ±0.3 | ±0.3 | ±0.3 | ±0.2 | ±0.2 | ±0.2 | ±0.3 | ±0.3 |
| HO8910 | 26.9 | 18.1 | 10.0 | 6.5 | 5.6 | 12.9 | 11.7 | 8.4 | 7.4 | 6.4 | 13.8 | 10.8 | 8.5 | 8.2 | 8.1 | 10.4 | 9.8 | 8.9 | 8.1 | 6.4 | 2.1 | 2.0 | 1.9 | 1.8 | 1.7 |
|  | ±0.5 | ±0.4 | ±0.4 | ±0.5 | ±0.4 | ±0.4 | ±0.4 | ±0.5 | ±0.5 | ±0.5 | ±0.3 | ±0.3 | ±0.3 | ±0.3 | ±0.3 | ±0.4 | ±0.3 | ±0.3 | ±0.3 | ±0.4 | ±0.3 | ±0.2 | ±0.2 | ±0.2 | ±0.2 |
| A2780 | 24.7 | 21.7 | 13.6 | 10.8 | 7.8 | 8.2 | 7.2 | 8.1 | 7.9 | 9.6 | 13.6 | 8.3 | 9.8 | 11.2 | 13.9 | 8.1 | 7.7 | 8.6 | 10.9 | 11.2 | 0.5 | 0.6 | 1.3 | 2.2 | 2.3 |
|  | ±0.4 | ±0.5 | ±0.4 | ±0.4 | ±0.5 | ±0.5 | ±0.3 | ±0.4 | ±0.3 | ±0.4 | ±0.5 | ±0.4 | ±0.4 | ±0.4 | ±0.5 | ±0.3 | ±0.3 | ±0.3 | ±0.3 | ±0.4 | ±0.1 | ±0.1 | ±0.2 | ±0.2 | ±0.2 |
| A2780DR | 176.1 | 73.6 | 36.3 | 28.0 | 20.2 | 104.1 | 115.8 | 53.3 | 58.2 | 59.1 | 76.5 | 34.3 | 42.2 | 54.8 | 88.6 | 36.3 | 43.2 | 47.1 | 50.6 | 64.3 | 3.6 | 3.9 | 3.2 | 4.9 | 3.9 |
|  | ±0.7 | ±0.6 | ±0.5 | ±0.5 | ±0.5 | ±0.6 | ±0.7 | ±0.5 | ±0.5 | ±0.5 | ±0.5 | ±0.4 | ±0.5 | ±0.4 | ±0.5 | ±0.5 | ±0.5 | ±0.4 | ±0.5 | ±0.5 | ±0.4 | ±0.4 | ±0.4 | ±0.5 | ±0.4 |
| * The data are presented as the means (upper panel) ±SD (lower panel); unit: μg/mL. Den1, 2000 mm^-2^; Den2, 1000 mm^-2^; Den3, 500 mm^-2^; Den4, 250 mm^-2^; Den5, 125 mm^-2^. | | | | | | | | | | | | | | | | | | | | | | | | | |

| **Table S7. Pearson’s correlation coefficient analysis of the linearity between cleaved caspase 3 levels and IC_50_ values measured by** **MTT, modified MTT, FCM, Trypan blue-based cell counting and limiting dilution assays.** | | | | | | | | | | |
| --- | --- | --- | --- | --- | --- | --- | --- | --- | --- | --- |
| **Forms of the relative levels of cleaved caspase 3 and the cell lines tested** | **MTT** | | **Modified MTT** | | **FCM** | | **Trypan blue** | | **Limiting dilution assay** | |
|  | r^2^ * | p value | r^2^ | p value | r^2^ | p value | r^2^ | p value | r^2^ | p value |
| Cleaved caspase-3/caspase-3 ratios | | | | | | | | | | |
| SKOV-3 | 0.184 | 0.472 | 0.450 | 0.215 | 0.746 | 0.059 | 0.673 | 0.089 | 0.982 | 0.001^#^ |
| ES-2 | 0.709 | 0.073 | 0.517 | 0.171 | 0.633 | 0.108 | 0.861 | 0.023^#^ | 0.850 | 0.026^#^ |
| HO8910 | 0.963 | 0.003^#^ | 0.935 | 0.007^#^ | 0.934 | 0.007^#^ | 0.674 | 0.089 | 0.828 | 0.032^#^ |
| A2780 | 0.741 | 0.061 | 0.102 | 0.600 | 0.062 | 0.686 | 0.322 | 0.319 | 0.525 | 0.166 |
| A2780DR | 0.084 | 0.637 | 0.510 | 0.175 | 0.054 | 0.706 | 0.001 | 0.961 | 0.940 | 0.006^#^ |
| Cleaved caspase-3/β-actin ratios | | | | | | | | | | |
| SKOV-3 | 0.486 | 0.191 | 0.668 | 0.095 | 0.901 | 0.014^#^ | 0.681 | 0.085 | 0.735 | 0.063 |
| ES-2 | 0.874 | 0.020^#^ | 0.791 | 0.044^#^ | 0.558 | 0.147 | 0.615 | 0.116 | 0.519 | 0.170 |
| HO8910 | 0.966 | 0.002^#^ | 0.929 | 0.007^#^ | 0.950 | 0.005^#^ | 0.737 | 0.069 | 0.888 | 0.017^#^ |
| A2780 | 0.860 | 0.023^#^ | 0.136 | 0.541 | 0.024 | 0.805 | 0.452 | 0.214 | 0.670 | 0.090 |
| A2780DR | 0.112 | 0.582 | 0.509 | 0.176 | 0.062 | 0.686 | 0.001 | 0.953 | 0.759 | 0.054 |
| * r^2^, square of the Pearson’s correlation coefficient.  # Statistical significance. | | | | | | | | | | |

| **Table S8. RT-PCR-measured inhibitory effects of siRNAs on target gene mRNA levels in ovarian cancer cells seeded at different densities.*** | | | | | | | | | | | | | | | | | | | | | | | | | |
| --- | --- | --- | --- | --- | --- | --- | --- | --- | --- | --- | --- | --- | --- | --- | --- | --- | --- | --- | --- | --- | --- | --- | --- | --- | --- |
| **Genes^#^** | **Cell lines** | **Seeding densities** | | | | | | | | | | | | | | | | | | | | | | | |
|  |  | **Den_1_ (2000/mm^2^)** | | | | **Den_2_ (1000/mm^2^)** | | | | | **Den_3_ (500/mm^2^)** | | | | | **Den_4_ (250/mm^2^)** | | | | | **Den_5_ (125/mm^2^)** | | | | |
|  |  | **siR1**^*^ | **siR2**^*^ | **NC** | **BC** | | **siR1** | **siR2** | **NC** | **BC** | | **siR1** | **siR2** | **NC** | **BC** | | **siR1** | **siR2** | **NC** | **BC** | | **siR1** | **siR2** | **NC** | **BC** |
| CDH1 | HO8910 | 0.3 | 0.2 | 1.2 | 1 | | 0.4 | 0.1 | 1.0 | 1 | | 0.3 | 0.2 | 0.9 | 1 | | 0.1 | 0.2 | 1.2 | 1 | | 0.2 | 0.3 | 1.1 | 1 |
| CTNNB1 | SKOV-3 | 0.2 | 0.1 | 0.9 | 1 | | 0.3 | 0.2 | 0.8 | 1 | | 0.1 | 0.1 | 0.8 | 1 | | 0.2 | 0.1 | 1.1 | 1 | | 0.3 | 0.1 | 0.7 | 1 |
|  | HO8910 | 0.2 | 0.4 | 1.1 | 1 | | 0.2 | 0.1 | 1.2 | 1 | | 0.1 | 0.3 | 0.9 | 1 | | 0.1 | 0.2 | 1.0 | 1 | | 0.1 | 0.1 | 1.2 | 1 |
| AKT1 | A2780 | 0.4 | 0.1 | 1.0 | 1 | | 0.1 | 0.2 | 1.1 | 1 | | 0.2 | 0.3 | 1.2 | 1 | | 0.1 | 0.2 | 1.1 | 1 | | 0.1 | 0.2 | 0.9 | 1 |
|  | A2780DR | 0.3 | 0.4 | 1.4 | 1 | | 0.3 | 0.3 | 1.2 | 1 | | 0.3 | 0.1 | 1.3 | 1 | | 0.1 | 0.1 | 1.2 | 1 | | 0.1 | 0.3 | 1.1 | 1 |
| AKT2 | A2780 | 0.2 | 0.3 | 0.9 | 1 | | 0.1 | 0.2 | 0.7 | 1 | | 0.1 | 0.2 | 0.8 | 1 | | 0.3 | 0.2 | 0.9 | 1 | | 0.1 | 0.2 | 0.9 | 1 |
|  | A2780DR | 0.1 | 0.1 | 0.8 | 1 | | 0.2 | 0.1 | 0.9 | 1 | | 0.2 | 0.3 | 1.1 | 1 | | 0.3 | 0.1 | 0.8 | 1 | | 0.1 | 0.3 | 0.9 | 1 |
| CDKN1B | ES-2 | 0.1 | 0.1 | 0.9 | 1 | | 0.1 | 0.1 | 0.9 | 1 | | 0.1 | 0.1 | 1.1 | 1 | | 0.1 | 0.1 | 0.9 | 1 | | 0.2 | 0.1 | 1.0 | 1 |
| BAD | A2780 | 0.1 | 0.2 | 0.8 | 1 | | 0.1 | 0.1 | 0.9 | 1 | | 0.2 | 0.1 | 0.9 | 1 | | 0.1 | 0.1 | 1.1 | 1 | | 0.3 | 0.2 | 1.1 | 1 |
|  | A2780DR | 0.1 | 0.2 | 1.0 | 1 | | 0.1 | 0.2 | 1.1 | 1 | | 0.3 | 0.2 | 1.3 | 1 | | 0.1 | 0.1 | 1.5 | 1 | | 0.1 | 0.2 | 1.1 | 1 |
| BCL2 | SKOV-3 | 0.1 | 0.1 | 0.8 | 1 | | 0.2 | 0.1 | 1.0 | 1 | | 0.1 | 0.2 | 1.0 | 1 | | 0.3 | 0.2 | 0.7 | 1 | | 0.1 | 0.2 | 0.9 | 1 |
|  | ES-2 | 0.3 | 0.1 | 0.9 | 1 | | 0.2 | 0.3 | 1.1 | 1 | | 0.3 | 0.2 | 1.1 | 1 | | 0.2 | 0.4 | 0.9 | 1 | | 0.4 | 0.3 | 1.1 | 1 |
| BAX | SKOV-3 | 0.4 | 0.3 | 1.1 | 1 | | 0.3 | 0.2 | 1.2 | 1 | | 0.1 | 0.3 | 1.3 | 1 | | 0.1 | 0.1 | 0.9 | 1 | | 0.3 | 0.2 | 1.1 | 1 |
| GJA1 | A2780 | 0.3 | 0.4 | 1.5 | 1 | | 0.1 | 0.2 | 1.1 | 1 | | 0.2 | 0.4 | 1.2 | 1 | | 0.1 | 0.3 | 1.3 | 1 | | 0.3 | 0.2 | 1.3 | 1 |
| * The data are presented as the means of three replicates of the cell transfection and RT-PCR experiments. siR1, siRNA No. 1; siR1, siRNA No. 2 (see Table S3); NC, siRNA negative control; BC, blank control.  # The full gene names represented by the listed abbreviations are the following (with brief descriptions):  CDH1, cadherin, type 1, also known as E-cadherin. This gene encodes a classical cadherin from the cadherin superfamily, and the encoded protein is a calcium-dependent cell-cell adhesion glycoprotein comprising five extracellular cadherin repeats, a transmembrane region and a highly conserved cytoplasmic tail. Mutations in this gene are correlated with gastric, breast, colorectal, thyroid and ovarian cancer. Loss of function is thought to contribute to cancer progression by increasing proliferation, invasion, and/or metastasis.  CTNNB1, cadherin-associated protein, beta 1, also known as β-catenin. The protein encoded by this gene is part of a complex of proteins that constitute adhering junctions, which anchor the actin cytoskeleton and might be responsible for transmitting the contact inhibition signal that causes cells to stop dividing once the epithelial sheet is complete. This protein also binds to the product of the APC gene. Mutations in this gene are a cause of colorectal cancer, pilomatrixoma, medulloblastoma and ovarian cancer.  AKT1/2, v-Akt murine thymoma viral oncogene homolog 1/2. AKT1 and its related homolog AKT2 are activated by binding to PIP_3_. The activation is rapid and specific and proceeds through phosphatidylinositol 3-kinase. Survival factors can suppress apoptosis in a transcription-independent manner by activating the serine/threonine kinase AKT1, which then phosphorylates and inactivates components of the apoptotic machinery. The gene family members have been shown to be amplified and overexpressed in ovarian cancer cell lines and primary ovarian tumors.  CDKN1B, cyclin-dependent kinase inhibitor 1B, also known as p27. This gene encodes a cyclin-dependent kinase inhibitor, which shares limited similarity with the CDK inhibitor CDKN1A/p21. The encoded protein binds to and prevents the activation of cyclin E-CDK2 or cyclin D-CDK4 complexes and thus control cell cycle progression at G1.  BAD, BCL2-associated agonist of cell death. The protein encoded by this gene is a member of the BCL-2 family. This protein positively regulates cell apoptosis by forming heterodimers with BCL-xL and BCL-2, thereby reversing their death repressor activity. The proapoptotic activity of this protein is regulated through phosphorylation by AKT and/or MAP kinases.  BCL2, B-cell CLL/lymphoma 2. This gene encodes an integral outer mitochondrial membrane protein that blocks apoptotic death.  BAX, BCL2-associated X protein. The protein encoded by this gene belongs to the BCL2 protein family. This protein is reported to interact with and increase the opening of the mitochondrial voltage-dependent anion channel (VDAC), which leads to loss of membrane potential and the release of cytochrome c. The expression of this gene is regulated by the tumor suppressor P53 and has been shown to be involved in P53-mediated apoptosis.  GJA1, gap junction protein, alpha 1, also known as connexin 43. This gene is a member of the connexin gene family. The encoded protein is a component of gap junctions, which are composed of arrays of intercellular channels that provide a route for the diffusion of low-molecular-weight materials from cell to cell.  (Functional descriptions of these genes were partially excerpted and/or modified from their corresponding entries under the “Gene” category at the website of the National Center for Biotechnology Information. http://www.ncbi.nlm.nih.gov). | | | | | | | | | | | | | | | | | | | | | | | | | |

|  |
| --- |
|  |
|  |

**Section V** – Clinical Data

| **Table S9. Demographic, clinical and pathological characteristics of the 112 previously treated ovarian cancer patients.** | | | | | |
| --- | --- | --- | --- | --- | --- |
| **Characteristics** | **Poor survival group*** (n = 63) | | | **Extended survival group*** (n = 49) | **p value** |
| *Demographic characteristics* | | | | | |
| Age |  | | |  | 0.196 |
| < 40 | 5 (7.9) | | | 3 (6.1) |  |
| 40-49 | 18 (28.6) | | | 14 (28.6) |  |
| 50-59 | 21 (33.3) | | | 11 (22.4) |  |
| 60-69 | 16 (25.4) | | | 12 (24.5) |  |
| ≥ 70 | 3 (4.8) | | | 9 (18.4) |  |
| Gravidity |  | | |  | 0.576 |
| 0-1 | 7 (11.1) | | | 5 (10.2) |  |
| 2-3 | 29 (46.0) | | | 19 (38.8) |  |
| 4-5 | 16 (25.4) | | | 11 (22.4) |  |
| ≥ 5 | 11 (17.5) | | | 14 (28.6) |  |
| Parity |  | | |  | 0.551 |
| 0-1 | 42 (66.7) | | | 30 (61.2) |  |
| 2-3 | 21 (33.3) | | | 19 (38.8) |  |
| Menopause |  | | |  | 0.789 |
| Yes | 37 (58.7) | | | 30 (61.2) |  |
| No | 26 (41.3) | | | 19 (38.8) |  |
| *Clinicopathological characteristics* | | | | | |
| Ascites |  | | |  | 0.382 |
| Yes | 10 (15.9) | | | 5 (10.2) |  |
| No | 53 (84.1) | | | 44 (89.8) |  |
| Peritoneal metastasis |  | | |  | <0.001^#^ |
| Yes | 25 (39.7) | | | 5 (10.2) |  |
| No | 38 (60.3) | | | 44 (89.8) |  |
| Lymphatic metastasis |  | | |  | 0.024^#^ |
| Yes | 19 (30.2) | | | 6 (12.2) |  |
| No | 44 (69.8) | | | 43 (87.8) |  |
| Histotype |  | | |  | 0.420 |
| Serous | 38 (60.3) | | | 26 (53.1) |  |
| Mucinous | 5 (7.9) | | | 9 (18.4) |  |
| Endometrioid | 9 (14.3) | | | 8 (16.3) |  |
| Clear cell | 7 (11.1) | | | 5 (10.2) |  |
| Undifferentiated | 4 (6.3) | | | 1 (2.0) |  |
| Stage |  | | |  | <0.001^#^ |
| I | 6 (9.5) | | | 21 (42.9) |  |
| II | 21 (33.3) | | | 20 (40.8) |  |
| III | 26 (41.3) | | | 7 (14.3) |  |
| IV | 10 (15.9) | | | 1 (2.0) |  |
| Grade |  | | |  | 0.076 |
| G1 | 9 (14.3) | | | 15 (30.6) |  |
| G2 | 28 (44.4) | | | 21 (42.9) |  |
| G3 | 26 (41.3) | | | 13 (26.5) |  |
| Size of residual site |  | | |  | 0.007^#^ |
| < 2 cm | 43 (68.3) | | | 44 (89.8) |  |
| ≥ 2 cm | 20 (31.7) | | | 5 (10.2) |  |
| Primary chemoresistance**  Yes  No | 19 (30.2)  44 (69.8) | | 5 (10.2)  44 (89.8) | | 0.011^#^ |
| Chemoresistant recurrence***  Yes  No | 63 (100)  0 (0) | | 29 (59.2)  20 (40.8) | | <0.001^#^ |
| *Microenvironmental pathological characteristics* | | | | | |
| Density of cancer cells  > median (6600 mm^-2^)  ≤ median | 29 (46.0)  34 (54.0) | | 27 (55.1)  22 (44.9) | | 0.446 |
| Density of stromal cells  > median (350 mm^-2^)  ≤ median | 33 (52.4)  30 (47.6) | | 24 (49.0)  25 (51.0) | | 0.849 |
| Density of M1 macrophages  > median (17 mm^-2^)  ≤ median | 33 (52.4)  30 (47.6) | | 24 (49.0)  25 (51.0) | | 0.849 |
| Density of M2 macrophages  > median (12 mm^-2^)  ≤ median | 33 (52.4)  30 (47.6) | | 23 (46.9)  26 (53.1) | | 0.703 |
| Density of CD4^+^ T cells  > median (6.3 mm^-2^)  ≤ median | 27 (42.9)  36 (57.1) | | 30 (61.2)  19 (38.8) | | 0.060 |
| Density of CD8^+^ T cells  > median (3.2 mm^-2^)  ≤ median | 20 (31.7)  43 (68.3) | | 26 (53.1)  23 (46.9) | | 0.033^#^ |
| *Molecular pathological characteristics* | | | | | |
| IHCpAkt+p62 score  > median (score=3)  ≤ median | 26 (41.3)  37 (58.7) | 10 (20.4)  39 (79.6) | | | 0.025^#^ |
| * The data are presented as numbers (%).  ** Primary chemoresistance is defined by the maximal diameter of the residual site shrinking by ≤50% or enlarging after a standard course of TP chemotherapy.  *** Chemoresistant recurrence indicates *in situ* and/or remote recurrence resistant to the standard TP chemotherapy. For the convenience of statistical analysis, patients with primary chemoresistance were also included in this category.  # Statistical significance: two-sided χ^2^ test; for 2×2 tables, Fisher’s exact test was applied. | | | | | |

| **Table S10. Univariate Cox regression analysis of 5-year progression-free and overall survival in 112 previously treated ovarian cancer patients.** | | | | |
| --- | --- | --- | --- | --- |
| **Characteristics** | **Progression-free survival** hazard ratio (95% CI)* | **p value** | **Overall survival** hazard ratio (95% CI) | **p value** |
| *Demographic characteristics* | | | | |
| Age |  | 0.741 |  | 0.391 |
| < 40 | 1.889 (0.744-4.801) |  | 3.511 (0.838-14.718) |  |
| 40-49 | 1.234 (0.591-2.573) |  | 3.217 (0.947-10.930) |  |
| 50-59 | 1.379 (0.665-2.857) |  | 3.366 (1.003-11.300) |  |
| 60-69 | 1.263 (0.596-2.673) |  | 3.256 (0.948-11.182) |  |
| ≥ 70 | 1 (reference) |  | 1 (reference) |  |
| Gravidity |  | 0.948 |  | 0.543 |
| 0-1 | 1.168 (0.546-2.496) |  | 1.557 (0.603-4.018) |  |
| 2-3 | 1.172 (0.684-2.011) |  | 1.672 (0.835-3.349) |  |
| 4-5 | 1.145 (0.628-2.084) |  | 1.509 (0.700-3.252) |  |
| ≥ 5 | 1 (reference) |  | 1 (reference) |  |
| Parity |  | 0.696 |  | 0.428 |
| 0-1 | 1.089 (0.711-1.668) |  | 1.236 (0.732-2.088) |  |
| 2-3 | 1 (reference) |  | 1 (reference) |  |
| Menopause |  | 0.623 |  | 0.641 |
| Yes | 0.901 (0.595-1.365) |  | 0.887 (0.537-1.466) |  |
| No | 1 (reference) |  | 1 (reference) |  |
| *Clinicopathological characteristics* | | | | |
| Ascites |  | 0.195 |  | 0.198 |
| Yes | 1.476 (0.820-2.657) |  | 1.560 (0.793-3.070) |  |
| No | 1 (reference) |  | 1 (reference) |  |
| Peritoneal metastasis |  | <0.001^#^ |  | <0.001^#^ |
| Yes | 0.431 (0.273-0.680) |  | 3.203 (1.924-5.333) |  |
| No | 1 (reference) |  | 1 (reference) |  |
| Lymphatic metastasis |  | 0.008 |  | 0.007^#^ |
| Yes | 0.521 (0.321-0.846) |  | 2.190 (1.273-3.767) |  |
| No | 1 (reference) |  | 1 (reference) |  |
| Histotype |  | 0.793 |  | 0.449 |
| Serous | 0.886 (0.320-2.456) |  | 0.609 (0.216-1.716) |  |
| Mucinous | 0.658 (0.209-2.075) |  | 0.291 (0.078-1.092) |  |
| Endometrioid | 0.764 (0.246-2.376) |  | 0.572 (0.175-1.867) |  |
| Clear cell | 1.085 (0.344-3.419) |  | 0.652 (0.190-2.237) |  |
| Undifferentiated | 1 (reference) |  | 1 (reference) |  |
| Stage |  | <0.001^#^ |  | <0.001^#^ |
| I | 0.102 (0.046-0.223) |  | 0.066 (0.024-0.185) |  |
| II | 0.152 (0.074-0.313) |  | 0.186 (0.086-0.399) |  |
| III | 0.346 (0.171-0.700) |  | 0.455 (0.218-0.949) |  |
| IV | 1 (reference) |  | 1 (reference) |  |
| Grade |  | 0.307 |  | 0.056 |
| G1 | 0.644 (0.362-1.146) |  | 0.394 (0.184-0.842) |  |
| G2 | 0.919 (0.581-1.456) |  | 0.779 (0.456-1.330) |  |
| G3 | 1 (reference) |  | 1 (reference) |  |
| Size of residual site |  | <0.001^#^ |  | <0.001^#^ |
| <2 cm | 0.419 (0.260-0.676) |  | 0.261 (0.153-0.445) |  |
| ≥2 cm | 1 (reference) |  | 1 (reference) |  |
| *Microenvironmental pathological characteristics* | | | | |
| Density of cancer cells  ≤ median (6600 mm^-2^)  > median | 1.377 (0.912-2.077)  1 (reference) | 0.128 | 1.248 (0.760-2.040)  1 (reference) | 0.382 |
| Density of stromal cells  ≤ median (350 mm^-2^)  > median | 0.839 (0.556-1.264)  1 (reference) | 0.400 | 0.920 (0.561-1.508)  1 (reference) | 0.740 |
| Density of M1 macrophages  ≤ median (17 mm^-2^)  > median | 1.034 (0.687-1.558)  1 (reference) | 0.871 | 0.889 (0.542-1.459)  1 (reference) | 0.642 |
| Density of M2 macrophages  ≤ median (12 mm^-2^)  > median | 0.893 (0.593-1.344)  1 (reference) | 0.587 | 0.778 (0.474-1.277)  1 (reference) | 0.321 |
| Density of CD4^+^ T cells  ≤ median (6.3 mm^-2^)  > median | 1.305 (0.865-1.969)  1 (reference) | 0.204 | 1.553 (0.941-2.560)  1 (reference) | 0.085 |
| Density of CD8^+^ T cells  ≤ median (3.2 mm^-2^)  > median | 1.532 (1.004-2.338)  1 (reference) | 0.048^#^ | 1.809 (1.063-3.078)  1 (reference) | 0.029^#^ |
| *Molecular pathological characteristics* | | | | |
| IHCpAkt+p62 score  ≤ median (score=3)  > median | 0.444 (0.289-0.681)  1 (reference) | <0.001^#^ | 0.370 (0.223-0.615)  1 (reference) | <0.001^#^ |
| * CI, confidence interval.  # Statistical significance: multivariate Cox regression analysis. | | | | |

| **Table S12. Multivariate Cox regression analysis of 5-year chemoresistant recurrence-free survival in 112 previously treated ovarian cancer patients** **(for all characteristics).*** | | | | |
| --- | --- | --- | --- | --- |
| **Characteristics** | ***In situ* recurrence- free survival** hazard ratio (95% CI)** | **p value** | **Remote recurrence-free survival** hazard ratio (95% CI) | **p value** |
| *Demographic characteristics* | | | | |
| Age |  | 0.285 |  | 0.210 |
| < 40 | 15.95 (0.572-444.5) |  | 0.871 (0.042-18.04) |  |
| 40-49 | 1.935 (0.310-12.08) |  | 2.851 (0.184-44.27) |  |
| 50-59 | 3.889 (0.594-25.48) |  | 7.211 (0.582-89.42) |  |
| 60-69 | 3.491 (0.646-18.88) |  | 2.400 (0.165-34.85) |  |
| ≥ 70 | 1 (reference) |  | 1 (reference) |  |
| Gravidity |  | 0.623 |  | 0.111 |
| 0-1 | 1.396 (0.216-9.005) |  | 3.830 (0.282-52.07) |  |
| 2-3 | 1.546 (0.370-6.459) |  | 1.010 (0.253-4.029) |  |
| 4-5 | 0.762 (0.175-3.314) |  | 6.843 (1.224-38.26) |  |
| ≥ 5 | 1 (reference) |  | 1 (reference) |  |
| Parity |  | 0.509 |  | 0.586 |
| 0-1 | 1.543 (0.426-5.593) |  | 1.394 (0.421-4.614) |  |
| 2-3 | 1 (reference) |  | 1 (reference) |  |
| Menopause |  | 0.280 |  | 0.037^#^ |
| Yes | 2.181 (0.530-8.968) |  | 5.656 (1.113-28.75) |  |
| No | 1 (reference) |  | 1 (reference) |  |
| *Clinicopathological characteristics* | | | | |
| Ascites |  | 0.035^#^ |  | 0.263 |
| Yes | 9.314 (1.169-74.23) |  | 2.788 (0.464-16.77) |  |
| No | 1 (reference) |  | 1 (reference) |  |
| Peritoneal metastasis |  | 0.003^#^ |  | 0.720 |
| Yes | 8.547 (2.088-35.71) |  | 0.667 (0.072-6.143) |  |
| No | 1 (reference) |  | 1 (reference) |  |
| Lymphatic metastasis |  | 0.540 |  | 0.177 |
| Yes | 1.686 (0.317-8.960) |  | 0.307 (0.055-1.703) |  |
| No | 1 (reference) |  | 1 (reference) |  |
| Histotype |  | 0.159 |  | 0.293 |
| Serous | 1.843 (0.096-35.43) |  | 0.084 (0.005-1.343) |  |
| Mucinous | 0.050 (0.001-1.860) |  | 0.097 (0.003-3.453) |  |
| Endometrioid | 1.266 (0.050-32.28) |  | 0.147 (0.006-3.620) |  |
| Clear cell | 1.990 (0.054-73.78) |  | 0.400 (0.015-10.89) |  |
| Undifferentiated | 1 (reference) |  | 1 (reference) |  |
| Stage |  | <0.001^#^ |  | 0.040^#^ |
| I | 0.002 (0.000-0.031) |  | 0.096 (0.002-5.535) |  |
| II | 0.010 (0.001-0.119) |  | 0.383 (0.014-10.73) |  |
| III | 0.084 (0.012-0.605) |  | 0.101 (0.009-1.091) |  |
| IV | 1 (reference) |  | 1 (reference) |  |
| Grade |  | 0.134 |  | 0.249 |
| G1 | 0.424 (0.089-2.030) |  | 1.403 (0.212-9.283) |  |
| G2 | 0.329 (0.106-1.019) |  | 0.280 (0.029-2.662) |  |
| G3 | 1 (reference) |  | 1 (reference) |  |
| Size of residual site |  | 0.401 |  | 0.111 |
| < 2 cm | 0.511 (0.107-2.444) |  | 4.059 (0.724-22.76) |  |
| ≥ 2 cm | 1 (reference) |  | 1 (reference) |  |
| *Microenvironmental pathological characteristics* | | | | |
| Density of stromal cells  ≤ median (350 mm^-2^)  > median | 3.375 (0.510-22.35)  1 (reference) | 0.207 | 0.009 (0.000-0.172)  1 (reference) | 0.002^#^ |
| Density of M1 macrophages  ≤ median (17 mm^-2^)  > median | 12.81 (1.969-83.33)  1 (reference) | 0.008^#^ | 11.25 (1.467-86.31)  1 (reference) | 0.020^#^ |
| Density of M2 macrophages  ≤ median (12 mm^-2^)  > median | 0.175 (0.023-1.323)  1 (reference) | 0.091 | 0.505 (0.135-1.886)  1 (reference) | 0.309 |
| Density of CD4^+^ T cells  ≤ median (6.3 mm^-2^)  > median | 1.119 (0.413-3.031)  1 (reference) | 0.825 | 3.421 (1-11.71)  1 (reference) | 0.050^#^ |
| Density of CD8^+^ T cells  ≤ median (3.2 mm^-2^)  > median | 5.839 (1.652-20.64)  1 (reference) | 0.006^#^ | 0.890 (0.350-2.264)  1 (reference) | 0.808 |
| *Molecular pathological characteristics* | | | | |
| IHCpAkt+p62 score  ≤ median (score=3)  > median | 0.113 (0.030-0.426)  1 (reference) | 0.001^#^ | 0.041 (0.009-0.184)  1 (reference) | <0.001^#^ |
| * Chemoresistant recurrence-free survival indicates two statuses: *in situ* and remote chemoresistant recurrence-free survivals. For the analysis of *in situ* chemoresistant recurrence-free survival, patients with a high cancer cell density (> 6600 mm^-2^) were enrolled; for the analysis of remote chemoresistant recurrence-free survival, patients with a low cancer cell density (≤ 6600 mm^-2^) were enrolled.  ** CI, confidence interval.  # Statistical significance: multivariate Cox regression analysis. | | | | |
